# The Effects of Selected Extraction Methods and Natural Deep Eutectic Solvents on the Recovery of Active Principles from *Aralia elata* var. *mandshurica* (Rupr. & Maxim.) J. Wen: A Non-Targeted Metabolomics Approach

**DOI:** 10.3390/ph17030355

**Published:** 2024-03-09

**Authors:** Alyona Kaleta, Nadezhda Frolova, Anastasia Orlova, Alena Soboleva, Natalia Osmolovskaya, Elena Flisyuk, Olga Pozharitskaya, Andrej Frolov, Alexander Shikov

**Affiliations:** 1 Department of Technology of Pharmaceutical Formulations, St. Petersburg State Chemical Pharmaceutical University, 197376 Saint-Petersburg, Russia; petrochenko.alyona@pharminnotech.com (A.K.); elena.flisyuk@pharminnotech.com (E.F.); 2 Laboratory of Analytical Biochemistry and Biotechnology, K.A. Timiryazev Institute of Plant Physiology RAS, 127276 Moscow, Russia; frolovanadja@yandex.ru (N.F.); orlova@ifr.moscow (A.O.); oriselle@yandex.ru (A.S.); 3 Department of Plant physiology and Biochemistry, St. Petersburg State University, Universitetskaya Nab. 7-9, 199034 St. Petersburg, Russia; natalia_osm@mail.ru; 4 Research Group of Biochemistry and Technology of Hydrobionts of Algae and Invertebrates, Murmansk Marine Biological Institute of the Russian Academy of Sciences (MMBI RAS), 17 Vladimirskaya Str., 183010 Murmansk, Russia; olgapozhar@mail.ru

**Keywords:** natural deep eutectic solvents, NADES, extraction, *Aralia elata*, non-targeted metabolics profiling, ultrasound-assisted extraction, vibrocavitation-assisted extraction

## Abstract

The methods and solvents employed in routine extraction protocols essentially impact the composition of the resulting extracts, i.e., the relative abundances of individual biologically active metabolites and the quality and stability of the isolates. Natural deep eutectic solvents (NADESs) represent a new class of environmentally friendly solvents, which are recognized as promising extractants alternative to conventional organic liquids. However, their relative efficiencies when applied in different extraction workflows are still poorly characterized. Therefore, here, we compare the potential of three extraction methods for the extraction of biologically active natural products from *Aralia elata* var. *mandshurica* with selected natural deep eutectic solvents (NADESs) using a non-targeted metabolomics approach**.** The non-targeted metabolite profiling relied on reversed-phase ultra-high-performance liquid chromatography–high-resolution mass spectrometry (RP-UHPLC-HR-MS). The roots of *A. elata* were extracted by maceration, ultrasound-assisted extraction (UAE), and vibrocavitation-assisted extraction (VAE). Principal component analysis (PCA) revealed a clear separation of the extracts obtained with the three extraction methods employed with NADES1 (choline chloride/malic acid) and NADES2 (sorbitol/malic acid/water). Based on the results of the hierarchical clustering analysis obtained for the normalized relative abundances of individual metabolites and further statistical evaluation with the *t*-test, it could be concluded that NADES1 showed superior extraction efficiency for all the protocols addressed. Therefore, this NADES was selected to compare the efficiencies of the three extraction methods in more detail. PCA followed by the *t*-test yielded only 3 metabolites that were more efficiently extracted by maceration, whereas 46 compounds were more abundant in the extracts obtained by VAE. When VAE and UAE were compared, 108 metabolites appeared to be more abundant in the extracts obtained by VAE, whereas only 1 metabolite was more efficiently recovered by UAE. These facts clearly indicate the advantage of the VAE method over maceration and UAE. Seven of the twenty-seven metabolites tentatively identified by tandem mass spectrometry (MS/MS) were found in the roots of *A. elata* for the first time. Additional studies are necessary to understand the applicability of VAE for the extraction of other plant materials.

## 1. Introduction

Extraction is the key step in the development of highly efficient phytopharmaceutical formulations. Solvents and extraction methods significantly impact the composition of the extracted metabolites, i.e., the quality and stability of the resulting isolates. 

Natural deep eutectic solvents (NADESs) represent a new class of environmentally friendly green solvents, which were recently proposed as an alternative to organic liquids. The philosophy of NADES application relies on the principles discovered by the Choi and Verpoorte group, who suggested that NADESs resemble the plant cellular medium for the biosynthesis of non-water-soluble small molecules and macromolecules [1]. 

It stands to reason to assume that this medium is the ideal solvent for the isolation of secondary metabolites from plant cells. Typically, the synthesis of NADESs relies on natural hydrogen donors and hydrogen acceptors taken in specific molar ratios [2]. To date, NADESs have been well established in natural product chemistry. Indeed, these solvents are currently successfully applied for the extraction of different classes of biologically active plant natural products [3,4,5]. They are featured with favorable biodegradability and biocompatibility, as well as low volatility, but they have high viscosity [2]. The latter factor might prevent a decrease in the mass transfer between the matrix and solvent. 

The conventionally used extraction method using maceration relies only on molecular diffusion, which might be the main explanation for its relatively low efficiency. Thus, the enhancement of the inter-phase mass transfer during the extraction process is necessary to increase the efficiency of metabolite diffusion from the plant cell to the solvent. This issue is especially important when highly viscous solvents are used. Therefore, several advanced extraction techniques, such as ultrasound-assisted extraction (UAE), supercritical fluid extraction, pulsed electric field-assisted extraction, extraction using rotary-pulsation apparatus (RPE), and vibrocavitation-assisted extraction (VAE), among others [6,7,8,9,10], were proposed for the enhancement of the extraction process in recent years.

Ultrasound-assisted extraction is based on the phenomenon of cavitation, i.e., the fact that cavitation bubbles are induced in liquids during the ultrasound treatment. The size of these bubbles increases during vacuum cycles, and they collapse when reaching a critical diameter. These collapses are accompanied by the release of enormous amounts of energy over short periods of time. Cavitation bubbles explode on the surface of the plant matrix particles, resulting in the efficient destruction of cell membranes and the extraction of intracellular secondary metabolites. This approach is widely recognized as an advantageous one. UAE in combination with NADES was efficiently employed for the extraction of saponin asiaticoside from *Centella asiatica* (L.) Urb. [11], triterpene saponins from *Trillium govanianum* Wall. ex D.Don [12], and *Polygonatum sibiricum* F.Delaroche [13], as well as different terpenoids from *Abelmoschus sagittifolius* Merr. [14].

As a further extension of this technology, vibration explosive installation was recently proposed for improving extraction efficiency using the vibrocavitation technique. This approach relies on the combination of the cavitation effect in the liquid phase with the intensive grinding of plant material due to cutting off plant particles in the gap between the rotor and the stator [15]. For example, the recovery of dioscin from the seeds of *Trigonella foenum graecum* L. increased in comparison to the maceration-based procedure when vibrocavitation-assisted extraction (VAE) was applied [16]. However, the efficiency of the vibrocavitation technique for the extraction of plant materials with NADESs has not been addressed so far.

*Aralia elata* var. *mandshurica* (Rupr. & Maxim.) J. Wen (syn. *A. elata*) is a medicinal plant rich in biologically active secondary metabolites, which are well known for their pharmacological properties. In Russia, the alcoholic tincture of *A. elata* roots is used in officinal medicine as an adaptogen to increase physical power and enhance resilience to stress, and it is considered a promising remedy in the treatment of fatigue [17,18,19,20,21,22]. To date, altogether, about three hundred biologically active metabolites have been identified in different *A. elata* isolates. These metabolites represent different classes such as triterpene saponins, terpenoids, flavonoids, organic acids and their esters, polyacetylenes, phenylpropanoids, and others [23,24,25]. The non-specific stress-protective activity of *A. elata* isolates is particularly associated with aralosides, which belong to the class of triterpene saponins. Thus, araloside A was shown to protect rats against the stress-induced ulcer [26]. The stress-protective activity of araloside C was manifested by the protection of H9c2 cardiomyoblasts against oxidative stress [27] and the prevention of hypoxia/reoxygenation-induced endoplasmic reticulum stress [28]. Furthermore, the administration of *A. elata* total araloside isolates to mice significantly reduced the phosphorylation rates of Jun N-terminal kinases, which are known to mediate cellular responses to intracellular and extracellular stressors [29].

According to the available literature data, NADESs were successfully implemented in the extraction of saponins [11,14,30,31]. Recently, we have reported the successful extraction of triterpene saponins from the roots of *A. elata* using a conventional maceration procedure, which involved using a broad selection of alternatively applied NADESs [24]. While we succeeded in identifying 20 individual triterpene saponins in NADES extracts, the non-specific metabolomics profiling of *A. elata* was not reported. Taking into account the well-established applicability of UAE and VAE for the enhancement of the extraction procedures, along with the high viscosity of NADESs, the implementation of these techniques for the extraction of plant metabolites from the roots of *A. elata* appears to be promising.

Therefore, in this study, we address the impact of extraction methods and selected NADESs on the recovery of biologically active natural products from *A. elata* using a non-targeted metabolite profiling approach. The results of this study reveal the characteristic patterns of prospective biologically active secondary metabolites that are extractable using different NADESs and extraction methods.

## 2. Results and Discussion

To investigate the secondary plant metabolome in the most efficient and comprehensive way, we performed our analyses both in the positive and negative ion modes, as specified in the Materials and Methods section. However, the signal intensities, observed in the analyses in the positive ion mode, were dramatically low in comparison to those detected in the negative ion mode. Therefore, here, we focus on the latter subset of the LC-MS-based information.

### 2.1. Comparison of the NADES Extraction Efficiencies and Extraction Method Performance

**Figure 1:**
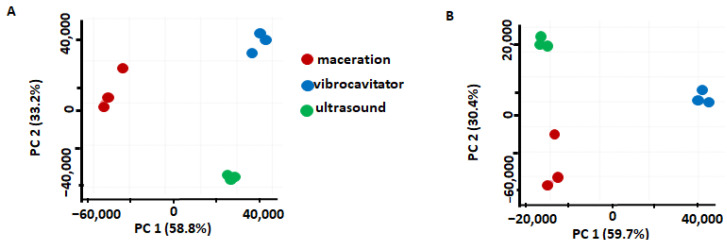
The results of the principal component analysis (PCA) with the score plots illustrating the comparisons of three different extraction methods (maceration, UAE, and VAE) in terms of their efficiencies observed with NADES1 (choline chloride/malic acid) (**A**) and NADES2 (sorbitol/malic acid (**B**).

In the first step, we compared three extraction methods (maceration, UAE, and VAE) applied with each NADES individually using an approach specific to that NADES. For this, principal component analysis (PCA) was employed. For NADES1 (choline chloride/malic acid (1:1)), PCA revealed a clear separation between the three different extraction methods in the corresponding score plot, where 58.8% and 33.2% of the total variance could be explained by the first and the second principal components, respectively (PC1 and PC2, Figure 1A). The PCA carried out for NADES2 (sorbitol/malic acid, 1:1 *w*/*w* with the supplementation of 10% (*w*/*w*) water) revealed a clear separation between the three extraction methods in the corresponding score plot, where 59.7% and 30.4% of the total variance could be explained by PC1 and PC2, respectively (Figure 1B). As can be seen from the first score plot (Figure 1A), the distribution of the individual samples obtained with NADES1 by VAE is relatively narrow, which might point to the high reproducibility of the results. However, as can be seen from Figure 1B, the maceration method showed the highest dispersion (i.e., the lowest reproducibility) when NADES2 was employed. 

In the next step, to gain a deeper insight into the relative extraction recoveries, the relative efficiencies of the considered extraction methods were investigated for both NADESs individually. For this, for each of the employed NADES, we performed paired comparisons for the three extraction methods—maceration, UAE, and VAE. 

#### 2.1.1. Comparison of Individual NADES in Terms of Their Efficiencies as the Extractants for Maceration

**Figure 2:**
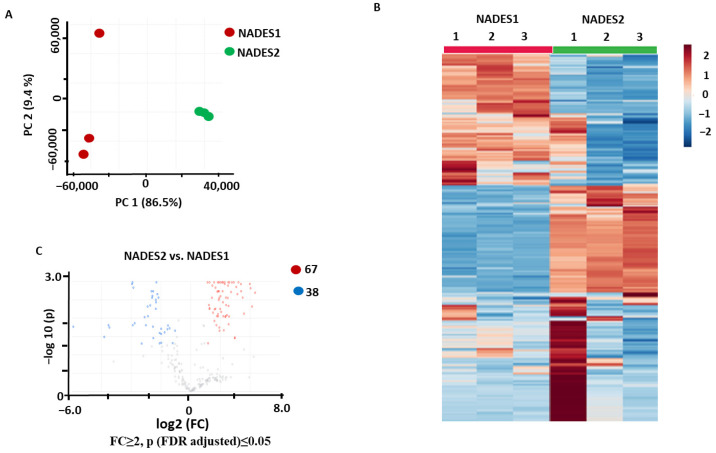
Comparison of the secondary metabolite profiles of the *Aralia elata* roots extracted using the maceration method with NADES1 and NADES2: results of the principal component analysis (PCA) with a score plot (**A**), hierarchical clustering analysis with a heatmap representation (**B**), and volcano plot with a graphical representation of differentially abundant analytes (**C**) with Benjamini–Hochberg false discovery rate (FDR) correction at *p* ≤ 0.05 and fold change (FC) ≥ 2. Color dots indicate metabolites showing statistically significant differences with FC ≥ 2 threshold level at *p* ≤ 0.05 compared to the controls. Thereby, the blue dots indicate metabolites (with the corresponding feature numbers) with increased contents in the extracts obtained with NADES2; the red dots indicate metabolites (with the corresponding feature numbers) with increased contents in the extracts obtained with NADES1. Metabolites indicated by gray dots showed no statistically significant differences.

The PCA results showed a clear separation between the two groups in the corresponding score plot, with 86.9% and 9.0% of the total variance explained by PC1 and PC2, respectively (Figure 2A). Hierarchical clustering analysis (HCA) with a heatmap repre sentation of the normalized relative abundances corresponding to the individual metabolites showed a relatively low level of intra-group variance and a clear separation of the two groups, which could be clearly seen from their separate clusterization (Figure 2B). The *t*-test analysis with a volcano plot representation (with a post-test Benjamini–Hochberg false discovery rate (FDR) correction at *p* ≤ 0.05 and fold change threshold of FC ≥ 2) yielded in total 105 differentially abundant metabolites, and of those, 38 and 67 features were more efficiently recovered using NADES2 and NADES1, respectively (Figure 2C). Thus, NADES1 showed better extraction efficiency when maceration was applied as the extraction method.

#### 2.1.2. Comparison of the NADES Efficiencies Observed with UAE

**Figure 3:**
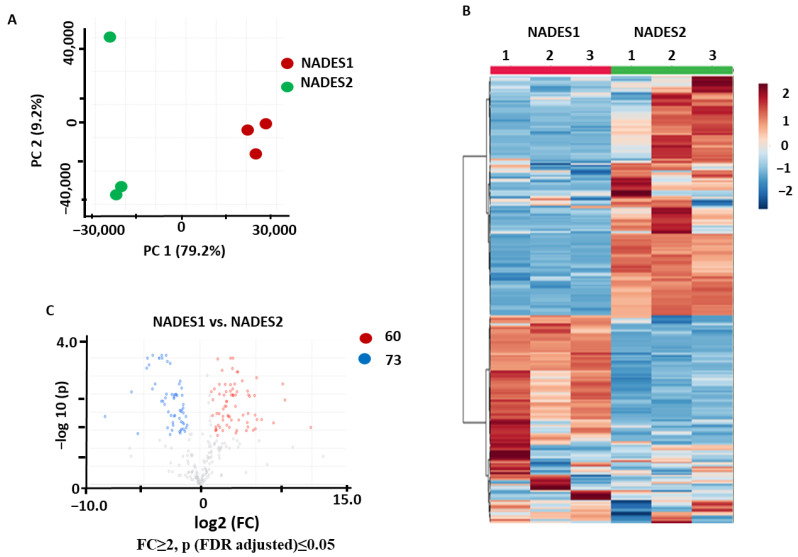
Comparison of the secondary metabolite profiles of the *Aralia elata* roots extracted by UAE with NADES1 and NADES2: results of the principal component analysis (PCA) with a score plot (**A**), hierarchical clustering analysis with a heatmap representation (**B**), and volcano plot with a graphical representation of differentially abundant analytes (**C**) with Benjamini–Hochberg false discovery rate (FDR) correction at *p* ≤ 0.05 and fold change (FC) ≥ 2. Color dots indicate the metabolites showing statistically significant differences with FC ≥ 2 threshold level at *p* ≤ 0.05 compared to the controls. Thereby, the blue dots indicate metabolites (with the corresponding feature numbers) with increased contents in the extracts obtained with NADES2; the red dots indicate the metabolites (with the corresponding feature numbers) with increased contents in the extracts obtained with NADES1. The metabolites marked with gray dots showed no statistically significant differences.

The PCA performed for the samples obtained using the UAE method showed a clear separation between the two groups in the corresponding score plot, with 79.2% and 9.2% of the total variance explained by PC1 and PC2, respectively (Figure 3A). Hierarchical clustering analysis with a heatmap representation of the normalized relative analyte abundances corresponding to the individual metabolites showed a relatively low level of intra-group variance, as well as a clear separation and independent clusterization of the two compared groups (Figure 3B). The *t*-test analysis with a volcano plot representation (Benjamini–Hochberg false discovery rate (FDR) correction at *p* ≤ 0.05 and FC ≥ 2) yielded 60 and 73 features that were more efficiently recovered with NADES1 and NADES2, respectively (Figure 3C).

The obtained data suggested that the efficiencies of both NADESs were comparable when UAE was employed as an extraction technique, although NADES2 appeared to be slightly more efficient than NADES1.

#### 2.1.3. Comparison of the NADES Efficiencies Observed with VAE

**Figure 4:**
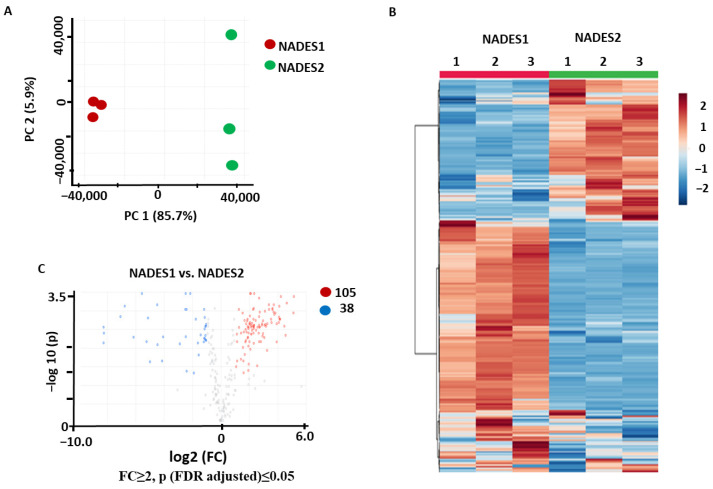
Comparison of the secondary metabolite profiles of the *Aralia elata* roots extracted using the VAE method with NADES1 and NADES2: the results of the principal component analysis (PCA) with a score plot (**A**), hierarchical clustering analysis with a heatmap representation (**B**), and volcano plot with a graphical representation of differentially abundant analytes (**C**) with Benjamini–Hochberg false discovery rate (FDR) correction at *p* ≤ 0.05 and fold change (FC) ≥ 2. Colored dots indicate the metabolites showing statistically significant differences with an FC ≥ 2 threshold at *p* ≤ 0.05 in comparison to the controls. Thereby, the blue dots indicate metabolites (with the corresponding feature numbers) with increased contents in the extracts obtained with NADES2, whereas the red dots indicate metabolites (with the corresponding feature numbers) with increased contents in the extracts obtained with NADES1. Metabolites marked with gray dots showed no statistically significant differences.

The PCA data acquired with the implementation of VAE showed a clear separation between the two groups in the corresponding score plot, with 85.7% and 5.9% of the total variance explained by PC1 and PC2, respectively (Figure 4A). Hierarchical clustering analysis with a heatmap representation of the normalized relative abundances corresponding to the individual metabolites showed a clear separation between the two sample groups (Figure 4B). The *t*-test analysis with a volcano plot representation (Benjamini–Hochberg false discovery rate (FDR) correction at *p* ≤ 0.05 and FC ≥ 2) yielded 105 and 38 features that were more efficiently recovered with NADES1 and NADES2, respectively (Figure 4C). This clearly indicated that NADES1 was more efficient when VAE was employed.

The clear group separation in the score plots (Figure 2A, Figure 3A and Figure 4A) and their distinctly separate clusterization in the corresponding heat maps (Figure 2B, Figure 3B and Figure 4B) indicated that different individual metabolites were more abundant when different solvents and extraction methods were used. This phenomenon can be explained by the fact that extraction efficiency strongly depends on the composition of NADESs. Due to the stimulation and inhibition of molecular interactions between the solvent and analyte molecules, the solvent can affect the solubility of the metabolite [32]. Both extracting solvents contained different hydrogen bond acceptors and the same component, malic acid, and these two-component systems adopt different spatial structures, which have pronounced affinity for mostly different metabolites. However, other factors cannot be excluded. Thus, another possible explanation might be the different physicochemical properties of solvents, such as polarity, viscosity, pH, etc. [33].

### 2.2. Comparison of the Extraction Methods

As can be seen from the previous section, NADES1 showed better performance based on the total number of efficiently extracted metabolites. Therefore, in the next step, we compared the efficiencies of the different extraction methods in combination with NADES1. As VAE appeared to be the most efficient extraction strategy, we assessed the efficiencies of maceration and UAE in paired comparisons with the samples obtained by VAE.

#### 2.2.1. Comparison of VAE and Maceration

**Figure 5:**
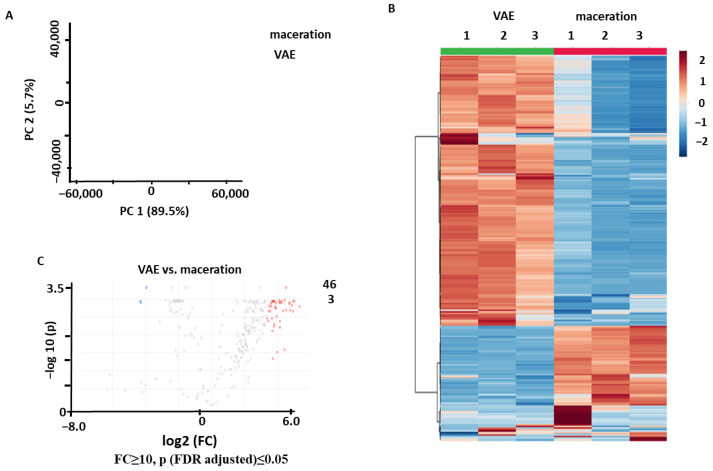
Comparison of the secondary metabolite profiles of the *Aralia elata* roots extracted with the VAE and maceration methods using NADES1: the results of the principal component analysis (PCA) with a score plot (**A**), hierarchical clustering analysis with a heatmap representation (**B**), and volcano plot with a graphical representation of differentially abundant analytes (**C**) with Benjamini–Hochberg false discovery rate (FDR) correction at *p* ≤ 0.05 and fold change (FC) ≥ 10. Colored dots indicate metabolites showing statistically significant differences with an FC ≥ 2 threshold at *p* ≤ 0.05 in comparison to the controls. Thereby, the blue dots indicate metabolites (with the corresponding feature numbers) with increased contents in the extracts obtained by maceration; the red dots indicate metabolites (with the corresponding feature numbers) with increased contents in the extracts obtained by VAE. Metabolites marked with gray dots showed no statistically significant differences.

The PCA performed for the samples obtained by VAE and maceration with NADES1 showed a good separation between the two groups in the corresponding score plot, with 89.5% and 5.7% of the total variance explained by PC1 and PC2, respectively (Figure 5A). Hierarchical clustering analysis (HCA) with a heatmap representation of the normalized relative abundances corresponding to the individual metabolites showed a clear separation between the analyzed groups (Figure 5B). The *t*-test analysis with a volcano plot representation (Benjamini–Hochberg false discovery rate (FDR) correction at *p* ≤ 0.05 and FC ≥ 10) yielded only 3 metabolites that were more efficiently extracted by maceration, whereas 46 compounds were more abundant in the extracts obtained by VAE (Figure 5C). Thus, VAE appeared to be the more efficient of the two extraction methods.

#### 2.2.2. Comparison of VAE and UAE

**Figure 6:**
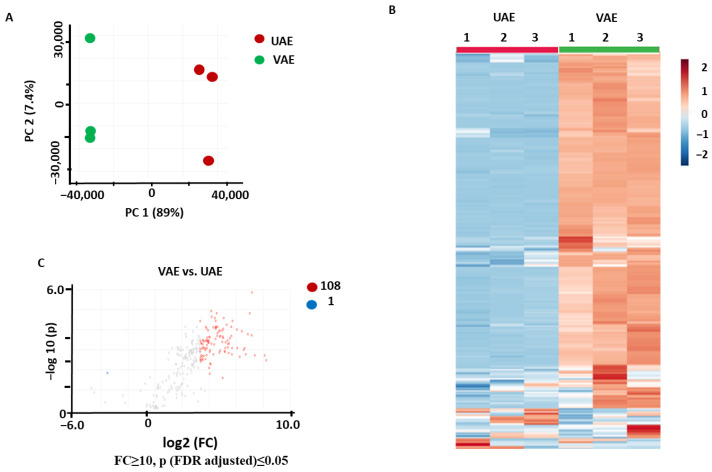
Comparison of the secondary metabolite profiles of the *Aralia elata* roots extracted with VAE and UAE methods using NADES1: the results of the principal component analysis (PCA) with a score plot (**A**), hierarchical clustering analysis with a heatmap representation (**B**), and volcano plot with a graphical representation of differentially abundant analytes (**C**) with Benjamini–Hochberg false discovery rate (FDR) correction at *p* ≤ 0.05 and fold change (FC) ≥ 10. Colored dots indicate the metabolites (with the corresponding feature numbers) showing statistically significant differences with an FC ≥ 2 threshold at *p* ≤ 0.05 in comparison to the controls. Thereby, the blue dots indicate the metabolites (with the corresponding feature numbers) with increased contents in the extracts obtained by UAE, whereas the red dots indicate metabolites with increased contents in the extracts obtained by VAE. The metabolites marked with gray dots showed no statistically significant differences.

The PCA performed for the samples that were obtained by VAE and UAE using NADES1 showed a clear separation between the two groups in the corresponding score plot, with 89% and 7.4% of the total variance explained by PC1 and PC2, respectively (Figure 6A). Hierarchical clustering analysis with a heatmap representation of the normalized relative abundances corresponding to the individual metabolites revealed a low level of intra-group variability and showed a clear separation of the analyzed groups (Figure 6B). The *t*-test analysis with a volcano plot representation (Benjamini–Hochberg false discovery rate (FDR) correction at *p* ≤ 0.05 and FC ≥ 10) yielded 108 metabolites more abundant in the extracts obtained by VAE and only 1 metabolite that was more efficiently recovered by UAE (Figure 6C). This fact clearly points to the advantage of the VAE method over UAE.

To explain the better efficiency of VAE in comparison to the maceration and UAE methods, it is necessary to understand the mechanisms behind these procedures. The main mechanism of maceration includes the diffusion of the extraction solvent in the cells, the desorption of cell metabolites, and the migration of the solute over the cell membrane and into the solvent around plant cells [10]. In the case of UAE, this mechanism is accompanied by the partial destruction of cells, capillary sound effects, acoustic microvortices, and local thermal effects [34]. UAE in combination with NADESs has been used for the extraction of soft plant parts such as flowers [35], powdered aerial plant parts [36], and leaves [37]. The roots of *A. elata* have a tough cellular structure that resists the destruction of cells by ultrasound and delays the penetration of viscous solvents. The mechanical degradation of the plant matrix is required for the intensification of the extraction. Decreasing particle size offers enhanced surface area, which facilitates the solvent’s entry into the plant cells. 

As can be seen from the data presented above, the VAE procedure showed the highest efficiency for the extraction of the *A. elata* roots in comparison to maceration and UAE (Figure 5 and Figure 6). The VAE procedure combines the ultrasonic effect and mechanical disintegration of plant material followed by the intensive circulation of the resulting low-disperse suspension with the solvent. Thereby, the particles of the plant material are crushed due to mechanical impact when passing through the working parts of the vibrocavitator. The temperature of the solutes located in the narrow gap between the working parts of the vibrocavitator locally increases. This leads to a decrease in NADES viscosity that is accompanied by the improved solubility of the extracted metabolites. 

Due to the partial mechanical destruction of the plant cells, the dissolved metabolites can be directly washed out from their cytoplasm, which further increases the extraction yields. Thus, the diffusion of the target metabolites through the cell membranes is accompanied by their elution from the destroyed cellular tissues. Obviously, the rates of both processes increase when a larger inter-phase contact surface can be achieved. In this case, solvent saturation with the active compounds can occur substantially faster. Hence, higher extraction yields can be achieved within shorter times. 

The resulting finely dispersed suspension of the plant material in the NADES is compressed at high speed in the extractor, and pressure increases in the compression and rarefaction zones. The excess pressure at the outlet of the working parts of the vibrocavitator is superimposed on the hydraulic circulation pressure in the extractor and reaches several atmospheres. During the rarefaction phase, cavities and cavitation bubbles form in the entire volume of the solvent, especially at the phase boundaries, i.e., in the locations where the liquid contacts with gas bubbles or/and tiny solid particles. When compressed again, these bubbles collapse, developing a pressure of up to hundreds of atmospheres, resulting in the formation of a highly intense shock wave. This leads to the additional mechanical destruction of solid plant particles and exudes small volumes of liquid from the phase interface, breaking up into small droplets and re-entering the plant [7,15,16]. This phenomenon helps to understand the high efficiency of NADES extraction using VAE. Similar mechanisms are implemented in rotary-pulsation extraction [10], which was successfully used for the extraction of hard plant materials such as seeds of *Pinus sibirica* Du Tour with oil [38] or the extraction of arabinogalactan from wood sawdust [39]. However, NADESs have not been tested in RPE thus far.

Thus, after the analysis of the acquired data, we can confidently conclude that the combined application of VAE and NADES1 is favorable for the extraction of secondary metabolites in *A. elata*.

### 2.3. Identification of Secondary Metabolites in the NADES Extracts from Aralia elata var. Mandshurica (Rupr. & Maxim.) J. Wen

#### 2.3.1. Identification of the Metabolites Annotated as Differentially Abundant in the NADES1 and NADES2 Extracts Obtained by Maceration

A comparison of the extraction efficiency for the maceration protocol based on the two different NADES revealed a total of 12 metabolites that could be annotated as differentially abundant with more than six-fold inter-group differences and the intensities of chromatographic signals exceeding 10^5^ counts per second (Table 1).

Compounds **1** and **2**, the relative abundances of which were lower in the extracts obtained with NADES1 in comparison to those obtained with NADES2, were tentatively annotated as malonyl hydroxydihydrocaffeoyl quinic acid and malonylcaffeoyl quinic acid based on the elemental compositions C_27_H_25_O_18_^–^ and C_20_H_21_O_13_^−^, as can be deduced from the signal of the [M-H]^−^ ion at *m*/*z* 487.1089 (calculated for C_27_H_25_O_18_^–^ at *m*/*z* 487.1093, 0.8 ppm, see Table 1) and [M-H] ^–^ ion at m/z 469.0987 (calculated for C_20_H_21_O_13_^−^ at *m*/*z* 469.0988, 0.3 ppm, see Table 1), respectively. The corresponding tandem mass spectra (MS/MS) revealed sequential losses of water, malonyl, and caffeic acid residues, resulting in the fragment ion signals at *m*/*z* 469.0923, *m*/*z* 353.0869, and *m*/*z* 191.0546 (Table 1, Figures S1–S3). 

The elemental composition of compound **3**, the relative abundance of which was higher in the extracts obtained with NADES1 compared to those prepared with NADES2, was attributed to the elemental composition C_22_H_29_O_14_^−^, as can be deduced from the signal of the [M-H]^−^ ion at *m*/*z* 517.1586 (calculated for C_22_H_29_O_14_^−^ at *m*/*z* 517.1563, −4.5 ppm, Table 1). Due to the presence of the fragment signal at *m*/*z* 353.0872 (loss of the fructofuranosyl moiety) and characteristic fragments at *m*/*z* 191.9559 (quinic acid), the compound was designated as pentofuranosyl dihydrocaffeoylquinic acid (Figure S4). 

Compound **4**, the relative abundance of which was higher in the extracts obtained with NADES1 in comparison to those prepared with NADES2, was annotated at *m*/*z* 577.1634. As the corresponding MS/MS spectra demonstrated characteristic fragments at *m*/*z* 181.0716, 261.0612, 279.0716, 297.0822, and 461.1507, this compound was annotated as a myo-inositol derivative (C_20_H_33_O_19_^−^, at *m*/*z* 705.1672, 2.1 ppm) (Table 1, Figure S5). 

Based on the signal of the [M-H]^−^ ion at *m*/*z* 225.0761, the elemental composition of compound **5**, the relative abundance of which was lower in the NADES1 extracts in comparison to those prepared with NADES2, was assigned to the elemental composition C_11_H_13_O_5_^−^ (calculated for C_11_H_13_O_5_^−^ at *m*/*z* 225.0768, 1.0 ppm, see Table 1). The corresponding MS/MS spectra, acquired at *m*/*z* 225.1, demonstrated characteristic fragments 107.0503, 137.0973, 163.0764, 181.0869, and 207.0660 based on which compound **5** was annotated as 3-hydroxy-5-(2-methoxy-1-methylethoxy)benzoate (Table 1, Figure S6). 

Based on its elemental composition (C_16_H_9_O_3_^−^, *m*/*z* 249.0557, 2.8 ppm), compound **6** was annotated as a malic acid derivative with the signal of [M-H]^−^ ion *m*/*z* 249.0550, as evidenced by the presence of the characteristic fragment signals at *m*/*z* 115.0038, 133.0143 (Table 1, Figure S7). The relative abundance of compound **7** was lower in the choline chloride–malic acid (NADES1) extracts in comparison to the NADES2 ones. 

The elemental composition of compound **7** was determined as C_32_H_33_O_18_^−^. This could be deduced from the signal of the [M-H]^−^ ion at *m*/*z* 705.1675 (calculated for C_32_H_33_O_18_^−^ at *m*/*z* 705.1672, −0.4 ppm, see Table 1). The presence of characteristic fragment signals at *m*/*z* 339.0503, 487.1212, and 513.1025 observed in the corresponding MS/MS spectrum led us to annotate this compound as a caffeoylquinic acid dimer, the relative abundance of which was higher in the NADES1 extracts (Table 1, Figure S8).

The elemental composition of compound **8**, the relative abundance of which was lower in the NADES1 extract, was determined as C_12_H_19_O_5_^−^, as could be deduced from the signal of the [M-H]^−^ ion at *m*/*z* 243.1237 (calculated for C_12_H_19_O_5_^−^ at *m*/*z* 243.1238, see Table 1). Due to the presence of the fragment signals at *m*/*z* 225.1129, *m*/*z* 207.1024, and *m*/*z* 181.1233 (sequential losses of water moieties) and the characteristic fragment at *m*/*z* 199.1338 (carbon dioxide moiety) in the corresponding MS/MS spectrum, this compound was designated as trihydroxy-dodecadienoic acid (Table 1, Figure S9). 

Compound **9**, which had a higher relative abundance in the NADES1 extract, was assigned to the elemental composition C_47_H_74_ClO_18_^−^, as could be deduced from the signal of the [MHCl-H]^−^ ion at *m*/*z* 961.4589 (calculated for C_47_H_74_ClO_18_^−^ at *m*/*z* 961.4569, see Table 1). The corresponding MS/MS spectra acquired at *m*/*z* 961.5 showed characteristic fragments at 565.3505, 631.3851, 733.5586, 763.4248, 793.4357, and 925.4802. Based on this fragmentation pattern, compound **9** was annotated as chikusetsusaponin IV (Table 1, Figure S10).

The elemental compositions of compounds **10** and **11** (with higher relative abundances in the NADES1 extracts) were determined as C_42_H_65_O_14_^−^ and C_41_H_63_O_13_^−^, respectively, as could be deduced from the signal of the [M-H]^−^ ion at *m*/*z* 793.4367 (calculated for C_42_H_65_O_14_^−^ at *m*/*z* 793.4380, see Table 1) and *m*/*z* 763.4286 (calculated for C_41_H_63_O_13_^−^ at *m*/*z* 763.4274, see Table 1), respectively. Due to the presence of the characteristic fragment signal at *m*/*z* 455.3522, corresponding to oleanolic acid, these compounds were assigned to the class of triterpenoid saponins. Furthermore, based on the characteristic MS/MS fragmentation patterns, compounds **10** and **11** were annotated as oleanolic acid hexuronide hexoside and pseudoginsenoside Rp1, respectively (Table 1, Figures S11 and S12). 

#### 2.3.2. Identification of the Metabolites Annotated as Differentially Abundant in the NADES1 Extracts Obtained by VAE and Maceration

NADES1 and VAE were identified as the most efficient combination of conditions for the extraction of the *A. elata* roots. In the comparison of the extracts obtained with the standard maceration procedure and VAE using NADES1, in total, 17 metabolites were discovered as differentially abundant with more than 10-fold inter-group differences and the intensities of the chromatographic signals exceeding 10^5^ counts per second (Table 2).

Compound **12** (with a lower relative abundance in the extracts obtained by VAE in comparison to those produced by maceration) was annotated as 5-malonyl-caffeoylquinic acid with the elemental composition C_20_H_21_O_1_^−^, as could be deduced from the signal of the [M-H]^−^ ion at *m*/*z* 469.0987 (calculated for C_20_H_21_O_13_^−^ at *m*/*z* 469.0988, 0.3 ppm, Table 2). CID-based MS/MS fragmentation in the linear ion trap (LIT) revealed a characteristic signal pattern, featured with sequential losses of malonyl and caffeic acid moieties, which yielded the characteristic fragment ion signals at *m*/*z* 353.0869 and *m*/*z* 191.0546 (Table 2, Figure S13). 

The elemental composition of compound **13** (more abundant in the extracts obtained by VAE) was attributed to C_54_H_84_ClO_25_^−^, as could be deduced from the signal of the [MHCl-H]- ion at *m*/*z* 1167.4970 (calculated for C_54_H_84_ClO_25_ at *m*/*z* 1167.4996, 2.2 ppm, Table 2). Due to the presence of the signals at *m*/*z* 697.3640, 969.4656, and 1131.5182 in the corresponding MS/MS spectrum, compound **13** was annotated as 3,16-dihydroxyolean-12-en-23,28-dioic acid 28-*O*-6-*O*-3-hydroxy-3-methylglutaryl)-hexopyranosyl-hexopyranosyl-hexopyranosyl ester (Table 2, Figure S14). 

The elemental composition of compound **14** (more abundant in the extracts obtained by VAE) was determined as C_53_H_82_ClO_24_^−^, based on the signal of the [MHCl-H]- ion at *m*/*z* 1137.4930 (calculated for C_53_H_82_ClO_24_^−^ at *m*/*z* 1137.4890, Table 2). The MS/MS spectra showed characteristic fragments at *m*/*z* 823.8554, 939.4536, 975.4535, and 1101.5084. Based on this signal pattern, compound **14** was annotated as celosin I (Table 2, Figure S15).

Compounds **15** (t**_R_** = 9.6 min) and **19** (t**_R_** = 11.3 min), which were more abundant in the extracts obtained by VAE, appeared to be the structural isomers and were attributed to the elemental composition C_48_H_74_ClO_20_^−^ based on the signals of the [MHCl-H]^–^ ions at *m*/*z* 1005.4430 (calculated for C_48_H_74_ClO_20_^–^ at *m*/*z* 1005.4467, 2.7–3.7 ppm, Table 2). The corresponding CID-MS/MS spectra showed characteristic fragments at *m*/*z* 688.4667, 730.2089, 842.4215, and 969.4651 for compound **15** and *m*/*z* 404.2202, 572.4813, 842.4193, 942.4771, and 969.4661 for compound **19**. Based on these data, compounds **15** and **19** were annotated as two 3-*O*-dihexopyranosyl-hexuronopyranosyl melilotigenin isomers (Table 2, Figures S16 and S22).

Compounds **16** (t**_R_** = 9.7 min), **20** (t**_R_** = 11.5 min), and **21** (t**_R_** = 11.6 min)**,** which were more abundant in the extracts obtained by VAE, also appeared to be structural isomers. Thereby, compounds **16** and **20** were assigned to the elemental composition C_48_H_76_ClO_20_^−^ (calculated for C_48_H_76_ClO_20_^−^ at *m*/*z* 1007.4624, 4.4 ppm, Table 2) based on the signals of the [MHCl-H]- ions at *m*/*z* 1007.4580, whereas compound **21** was assigned to the elemental composition C_48_H_75_O_20_^−^ (calculated for C_48_H_75_O_20_^−^ at *m*/*z* 971.4857, 1.6 ppm, Table 1) based on the signal of the [M-H]^−^ ion at *m*/*z* 971.4841. The corresponding CID-MS/MS spectra revealed characteristic fragment signals at *m*/*z* 777.4055, 799.3860, 911.4629, 939.4566, and 971.4799 for compounds **16** and **20** and characteristic fragments at *m*/*z* 407.3312, 471.3464, 567.3672, 747.4294, 790.4078, 809.4285, and 925.4777 for compound **21**. Based on these patterns, compounds **16**, **20,** and **21** were annotated as three sophoraflavoside II isomers (Table 2, Figures S17, S23–S25).

Compounds **17** (t**_R_** = 9.8 min) and **22** (t**_R_** = 12.1 min)**,** which were more abundant in the extracts obtained by VAE, also appeared to be structural isomers. Both compounds were assigned to the elemental composition C_47_H_73_O_19_^−^ (calculated for C_47_H_73_O_19_**^−^** at *m*/*z* 941.4752, 4.8 ppm, Table 2) based on the signal of the [M-H]^−^ ion at *m*/*z* 941.4707. The corresponding CID-MS/MS spectra demonstrated characteristic fragments at *m*/*z* 465.3363, 537.3576, 583.3629, 627.3525, 669.3629, 733.4151, 777.4045, and 819.4150 for compound **17** and *m*/*z* 565.3526, 609.3419, 745.4152, 777.4048, and 807.4152 for compound **22**. Based on these patterns, compounds **17** and **22** were annotated as two dihydrogypsogenin 3-*O*-pentopyranosyl-hexpyranoxyl-hexuronopyranoside isomers (Table 2, Figures S18, S19, S26 and S27).

Compounds **18** (t**_R_** = 10.5 min), **23** (t**_R_** = 12.3 min), **26** (t**_R_** = 12.8 min), and **27** (t**_R_** = 12.9 min), which were more abundant in the extracts obtained by VAE, are representatives of the group of triterpenoid saponins. All these compounds demonstrated a characteristic fragment at *m*/*z* 455.3522 in their MS/MS spectra, based on which all four were classified as triterpenoid saponins, specifically, derivatives of oleanolic acid. The elemental composition of compound **18** was determined as C_48_H_75_O_19_^−^, as could be deduced from the signal of the [M-H]^−^ ion at *m*/*z* 955.4914 (calculated for C_48_H_75_O_19_^−^ at *m*/*z* 955.4908, Table 1). Based on this, compound **18** was tentatively annotated as calendulaglycoside C (Table 2, Figures S20 and S21). The elemental composition of compound **23** was determined as C_47_H_74_ClO_18_^−^ based on the signal of the [MHCl-H]- ion at *m*/*z* 961.4589 (calculated for C_47_H_74_ClO_18_^−^ at *m*/*z* 961.4569, Table 1). Therefore, compound **23** was tentatively identified as chikusetsusaponin IV (Figure S28). The elemental composition of compound **26** was designated as C_42_H_65_O_14_^−^ based on the signal of the [M-H]^−^ ion at *m*/*z* 793.4367 (calculated for C_42_H_65_O_14_^−^ at *m*/*z* 793.4380, Table 1). Thus, compound **26** was tentatively annotated as oleanolic acid hexoside–hexuronide (Figures S32 and S33). The elemental composition of compound **27** was determined as C_41_H_63_O_14_^−^, as could be deduced from the signal of the [MHCl-H]- ion at *m*/*z* 779.4216 (calculated for C_41_H_63_O_14_^−^ at *m*/*z* 779.4223, Table 1). Based on this fragmentation pattern, compound **27** was tentatively annotated as 3-*O*-(pentopyranosyl-hexuronopyranosyl) hederagenin (Table 2, Figure S34).

Compound **24** (more abundant in the extracts obtained by VAE) was assigned to the elemental composition C_42_H_66_ClO_15_^−^ (calculated for C_42_H_66_ClO_15_^−^ at *m*/*z* 845.4096, see Table 1), which could be derived from the signal of the [MHCl-H]- ion at *m*/*z* 845.4110. The corresponding CID-MS/MS spectra showed characteristic fragments at *m*/*z* 407.3310, 477.3728, 539.3671, 567.3671, 587.3570, 647.3779, 747.4291, and 809.4301. Based on this pattern, compound **24** was tentatively annotated as ilexoside XLVIII (Table 2, Figures S29 and S30).

Compound **25** (more abundant in the extracts obtained by VAE) was assigned to the elemental composition C_41_H_61_O_14_^−^ (calculated for C_41_H_61_O_14_^−^ at *m*/*z* 777.4067, see Table 1), which could be derived from the signal of the [M-H]^−^ ion at *m*/*z* 777.4057. The corresponding CID-MS/MS spectra demonstrated a characteristic pattern of fragment signals at *m*/*z* 469.3318, 565.3526, 583.3625, 627.3519, 645.3624, and 777.4057. Based on this pattern, compound **25** was tentatively annotated as 3-*O*-hexopyranosyl-pentopyranosylurs- 12,18-diene-24,28-dioic acid (Table 2, Figure S31).

In terms of fold changes, according to Table 2, compounds **13–28** turned out to be 1.3^2^–2.9^2^ times higher in abundance when using VAE, while compounds 12 were 2.1^2^–2.9^2^ times higher when using maceration.

### 2.4. The Cross-Validation of the Quantitative Results Using the Targeted Relative Quantification Strategy

**Figure 7:**
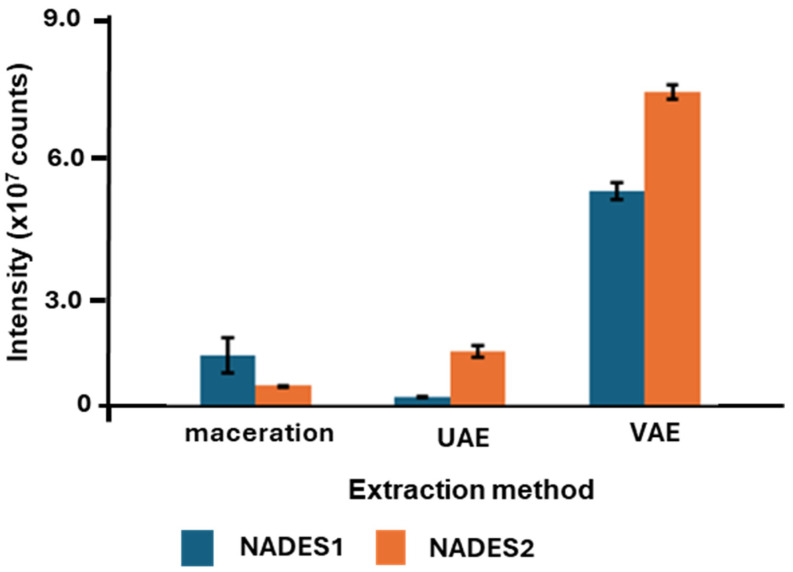
Relative total abundances (assessed as the MS-signal intensities) of aralosides A, B, and C in the extracts obtained by maceration, ultrasound (UAE), and vibrocavitation (VAE). For this, the compound-specific extracted ion chromatograms (XICs) were generated at *m*/*z* 925.4796, *m*/*z* 1057.5255, and *m*/*z* 1087.5308 for aralosides A, B, and C, respectively, and the characteristic chromatographic peaks were integrated at the t_R_s values of 10.6, 10.6, and 10.3, respectively.

To verify the validity of the results obtained by the non-targeted metabolomics approach, we decided to cross-validate our results using the targeted quantification of well-known components of the aralia root extracts. As aralosides A, B, and C are recognized marker compounds and biologically active constituents of *Aralia*, which are recommended by Russian and Belorussian Pharmacopoeias for the quality control of *A. elata* medicinal formulations [40], these compounds appear quite suitable for this type of validation. Therefore, we generated extracted ion chromatograms (XICs, *m*/*z* ± 0.02) for the signals at *m*/*z* 925.4796, *m*/*z* 1057.5255, and *m*/*z* 1087.5308, corresponding to the [M-H]^−^ ions of aralosides A, B and C, respectively, and integrated their characteristic chromatographic peaks at the t_Rs_ of 10.6, 10.6, and 10.3, respectively (which were assigned based on the additionally acquired MS/MS spectra). As these three metabolites are featured with high structure similarity, they can be expected to have quite similar ionization efficiencies. Therefore, we compared the sums of their signal intensities in the extracts obtained with the maceration, UAE, and VAE methods using different NADESs. The total intensity values appeared to significantly vary when different extraction methods were applied, whereas the differences attributable to the NADES nature were less pronounced, although significant. In this experiment, VAE was confirmed as the most efficient extraction method for the recovery of the three marker aralosides, whereas maceration and UAE were generally comparable in their performance (Figure 7).

The major compound class whose recovery was significantly affected by extraction conditions was saponins. Twelve representatives of this group (namely compounds **9–11**, **13, 15, 17, 19, 22, 23, 25, 26,** and **27**) were reliably identified in the *A. elata* roots based on characteristic MS/MS patterns (Table 1 and Table 2). The other group of the identified bioactive natural products was represented by phenylpropanoids, namely five caffeoylquinic acids (CQAs; compounds **1, 2, 3, 7,** and **12**). In plants, CQAs act as protectors, underlying their tolerance to biotic or abiotic stress. Consumption of these plant metabolites by humans is associated with a wide range of potential benefits with well-defined therapeutic applications. These applications are typically characterized by the pronounced antioxidant, antibacterial, antiparasitic, neuroprotective, anti-inflammatory, anticancer, antiviral, and antidiabetic effects of CQAs [41]. 

Despite the strong predominance of saponins and phenylpropanoids among the identified compounds, some minor contributors need to be mentioned. Thus, myo-inositol derivatives, the representative of which (**4**) was identified here as preferably extracted with NADES1 using maceration protocol (Table 1), are known to be involved in the regulation of reactive oxygen species (ROS) production and the modulation of signal transduction [42]. Compounds **9** and **23**, which were attributed to chikusetsusaponin IV (syn. araloside A) and more efficiently extracted with NADES1 using VAE, showed anticancer effects, which relied on the inhibition of cell proliferation, the retardation of cell cycle arrest, and the induction of cell apoptosis [43]. On the other hand, terpenoid 3-hydroxy-5-(2-methoxy-1-methylethoxy)benzoate (**5**), which was recovered in higher amounts using the maceration procedure with NADES2, showed antioxidant and antiglycation properties [44]. The compounds annotated as oleanolic acid hexuronide–hexoside (**10** and **26**), more efficiently extracted with NADES1 using VAE, and pseudoginsenoside Rp1 (**11**), more efficiently extracted with NADES1 in comparison to NADES2, are known as saponins with pronounced anticancer and anti-inflammatory activities [45]. Not less importantly, hepatoprotective effects and lipid-lowering activity were previously reported for compound **14**, which was more efficiently extracted with NADES1 using VAE [46]. Finally, some other compounds detected as more represented in extracts obtained using the VAE procedure and NADES1 are known for their biological activity. Thus, calendulaglycoside C (**18**) is associated with antitumor, anti-inflammatory, and antioedematous activities [47]. The derivatives of melilotigenin (**15,19**) were suggested to have anticancer activity [48]. Ilexoside XLVIII (**24**) exhibited inhibitory activity on acyl CoA cholesteryl acyl transferase [49]. For compound **27**, a derivative of hederagenin, some antitumor activity was observed [50]. Importantly, as far as we know, seven of these differentially abundant compounds (**4, 8, 14, 16, 20, 21,** and **25**) were tentatively identified in *Aralia elata* roots for the first time. 

## 3. Materials and Methods

### 3.1. Chemicals

The components of NADES were obtained from the following manufacturers: NevaReactive (Saint Petersburg, Russia) (choline chloride (≥99%), sorbitol (≥99%), and malic acid (≥99%)). Reagents for UHPLC-MS were used from Honeywell Riedel-de Haen, namely acetonitrile (LC-MS grade) and methanol (LC-MS grade); Merck KGaA (Darmstadt, Germany) provided formic acid (for LC-MS, ≥98%); Sigma-Aldrich (Saint Louis, MO, USA) provided ammonium formate (for LC-MS, ≥99.0%). Water was purified in-house on a water conditioning and purification system, namely Barnstead GenPure Pro UV-TOC (resistance 18.2 mΩ·cm, Thermo Fisher Scientific, Sweden).

### 3.2. Raw Material of Plants and the Composition and Preparation of NADESs

The roots of *A. elata* were obtained from a private supplier from the Far East (Khabarovsk Region) of Russia. The samples were characterized and deposited in the Department of Pharmacognosy of the Saint Petersburg State Chemical Pharmaceutical University (voucher of specimens MW0107420). *A. elata* roots were ground using a disk mill and stored in a dry place before extraction using NADESs. 

Two NADESs, namely NADES1, comprising choline chloride/malic acid (1:1), and NADES2, comprising sorbitol/malic acid (1:1 *w*/*w*) supplemented with water (10% *w*/*w* water), were selected according to the results of our previous study [24]. The NADESs were prepared using the heating method with continuous stirring [2]. The ratio of the raw plant material and the NADES employed for the extraction was 1:40 (*w*/*v*). 

### 3.3. Extraction Procedures

**Figure 8:**
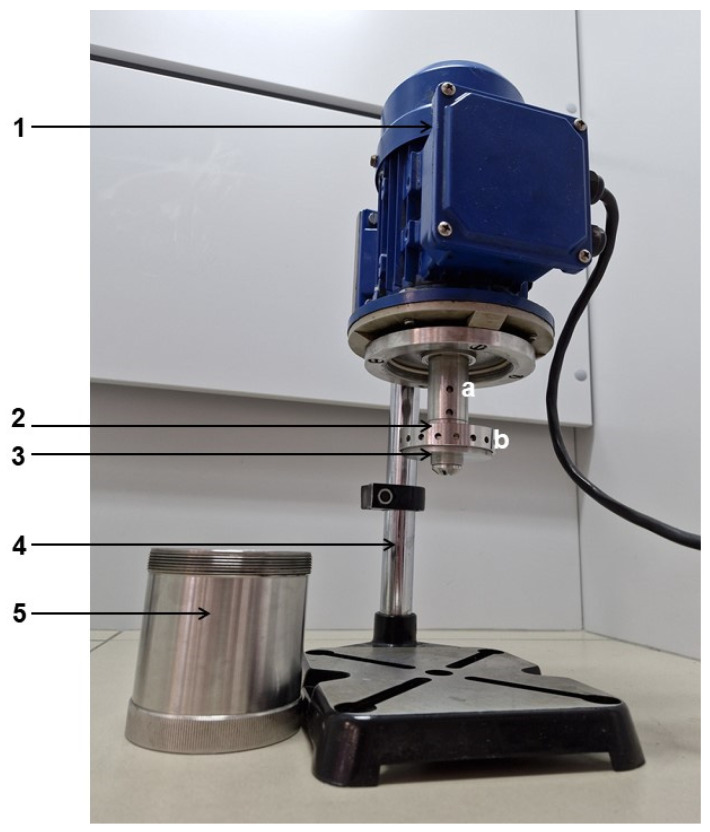
The design of the vibrocavitator: electric motor (1); stator (2); rotor (3); holder (4); extraction cup (5); stator holes (a and b).

The dry roots of *A. elata* were extracted using three methods. Maceration was performed at 60 °C with continuous stirring at 300 rpm for 60 min. Ultrasound-assisted extraction (UAE) was carried out in an ultrasound bath (Sapfir, UZV-3,8, Moscow, Russia) operated at 35 kHz and a temperature of 40 °C for 45 min. Before vibrocavitation-assisted extraction, the plant material was soaked with the NADES for 15 min and then transferred to the extractor. The vibrocavitator (laboratory sample of the device developed at St. Petersburg State Technical University, Russia) was applied at 50 Hz for 5 min [16]. The corresponding instrumentation is presented in Figure 8.

The workflow of vibrocavitation-assisted extraction was as follows: First, the plant material was supplemented with the NADES and transferred to the extraction cup (5). Then, the extraction cup was connected with the motor (1) via a threaded connection to ensure that both the stator and the rotor were inside the cup and were in direct contact with the plant material suspension in the NADES. When the instrument was operated, the suspension entered (due to centrifugal forces) through the hole (a) into the stator rod (3) and exited out of the hole (b) as well as the gap between the stator and the rotor in the bottom part of the stator. The plant material was additionally ground in the gap between the stator and the rotor. The resulting extracts obtained using all extraction methods were filtered, and the liquid phase (2.0 g of NADES extracts) was dissolved in 3 mL of water. Afterward, aliquots of 200 µL in volume were used for the UHPLC analysis. All analyses were performed in triplicate.

### 3.4. RP-UHPLC-ESI-LIT-Orbitrap-MS/MS

Extracts (5 µL) were separated at a flow rate of 0.4 mL/min on an EC 150/2 Nucleoshell RP18 column (end-caped C18 phase, ID 2 mm, length 150 mm, particle size 2.7 μm, Macherey Nagel, Düren, Germany) at 40 °C using a Dionex UltiMate 3000 UHPLC system (Thermo Fisher Scientific, Bremen, Germany) coupled online to a hybrid LTQ-Orbitrap Elite mass spectrometer via a HESI source (Thermo Fisher Scientific, Bremen, Germany) at 300 °C. Eluents A and B were 0.3 mmol/L ammonium formate (adjusted to pH 3.5 with formic acid) and acetonitrile, respectively. After a 2 min isocratic step (5% eluent B), the analytes were eluted in a 17 min linear gradient to 95% eluent B. 

The column effluents were introduced online in an Orbitrap Elite mass spectrometer operated in the negative and positive ion modes. The analysis in the negative mode was performed under an ion spray voltage of 3.8 kV, with the nebulizer and auxiliary gases set to 20 and 10 psig, respectively. The capillary temperature was set to 275 °C. The analysis in the positive mode was performed under an ion spray voltage of 4.0 kV, with the nebulizer and auxiliary gases set to 25 and 21 psig, respectively. The capillary temperature was set to 325 °C. 

Analytes were annotated in preliminary data-dependent acquisition experiments designed according to the doubly play algorithm, where a survey Orbitrap-MS scan with a mass resolution of 30,000 and MS/MS scans for the three most abundant signals selected in the survey were run. Collision-induced fragmentation (CID) was performed in a linear ion trap via resonance activation (30% normalized collision energy) in the presence of He as a collision/cooling gas. The corresponding quasi-molecular ions were isolated with a width of 2 *m*/*z*, and the activation time and relative activation frequency were 10 ms and 0.250, respectively.

### 3.5. Analysis of Secondary Metabolites in the Aralia elata Root Extracts

Prior to the analysis of the experimental samples, an optimization procedure was carried out with a set of test samples. Thereby, the optimal injection volumes of the aralia root extracts could be determined. The instrumental analysis sequence included randomly ranked experimental samples; 5 quality controls (QCs), representing a mixture of samples (20 µL aliquots); and blank samples. External QCs were injected after every nine samples. The analysis relied on a non-targeted metabolomics strategy, which involved using a combination of MS1-only profiling UHPLC-MS scans and UHPLC-MS/MS scans performed as data-dependent acquisition (DDA) experiments. Thereby, negatively and positively charged quasi-molecular metabolite ions, [M−X]^−^ and [M+X]^+^ (where X is single-charged cations), were recorded. The quality of chromatograms was assessed using Thermo Scientific™ Xcalibur™ Software 2.2 SP1.48. Chromatogram processing, involving the alignment of chromatograms with the retention time (t_R_) of analytes, marking chromatographic peaks, the deconvolution of mass spectra, and the integration of analyte peak areas from chromatograms reconstructed by *m*/*z* and t_R_ values of quasi-molecular ion, was performed in MSDial 4.9 Software (http://prime.psc.riken.jp/compms/msdial/main.html); parameter settings of the method are shown in Supplement S2 (Table S1). The relevance of the selected parameters was confirmed through a parallel comparison of integration results (namely fold changes) of individual peaks in Thermo Scientific™ Xcalibur™ Software. The selected metabolites with established t_R_ and *m*/*z* with a signal intensity of more than 10^5^ were independently integrated using Xcalibur Software, and fold changes of the corresponding integrated peak areas of the sample groups corresponding to different extraction setups were compared with the corresponding ones obtained using the MSDial method. Based on the obtained data, namely the correspondence of fold changes, it was concluded that the selected MSDial settings were correct. The corresponding fold changes obtained with different methods demonstrated the adequacy of the analysis method in pairwise comparisons (Table S2).

During the processing of the chromatography–mass spectrometric information of the *Aralia elata* extracts, the integrated peak areas of the ions were automatically determined from all samples and organized as a digital matrix. Furthermore, successful data normalization to the original extract concentrations (g of original lyophilized material/L of the extractant) prepared with different extraction workflows was verified through the assessment of the RSD values, which appeared to be within 5% (Tables S3 and S4). Therefore, our matrix was considered to be valid and applicable for further analysis. Considering this, the matrix was filtered to exclude samples from the statistical analysis according to the RSD of the quality controls (samples with RSD of QCs ≥30% were excluded), and analyte ions were not detected in ≥20% of samples; the imputation of the missing values was performed using KNN (sample-wise) manner [51]. Metaboanalyst 5.0 software (https://www.metaboanalyst.ca/) was used for metabolomics data analysis via principal component analysis (PCA), hierarchical cluster analysis with heatmap representation, and *t*-test analysis with a volcano plot representation (Benjamini–Hochberg false discovery rate (FDR) correction at *p* ≤ 0.05; FC ≥ 2 and FC ≥ 10) to visualize statistically significant changes in relative abundances of individual metabolites.

The cross-validation of the method standardization was accomplished with three aralosides A, B, and C, previously identified for Aralia (Table S5) [24].

## 4. Conclusions

In this study, the extraction of secondary metabolites from *A. elata* roots was investigated using two NADESs and three extraction techniques—maceration, UAE, and VAE. Thereby, the efficiencies of these extraction setups were compared at the level of multiple individual metabolites. To the best of our knowledge, this is the first study using the non-targeted metabolomics approach (metabolite profiling) to assess this aspect. Based on the results of the principal component analysis and hierarchical clustering analysis, NADES1 was found to be the most efficient extractant, and VAE showed the highest extraction efficiency with the roots of *A. elata* compared to maceration and UAE. In total, 27 highly abundant metabolites were tentatively identified in the roots of *A. elata* by RP-UHPLC-ESI-LIT-Orbitrap-MS/MS. Seven of them were found in *A. elata* roots for the first time. Our results indicate the high efficiency of VAE for the extraction of multiple metabolites from hard plant materials. Obviously, this approach is promising for further optimization and improvement in terms of better recoveries of biologically active natural products.

## Figures and Tables

**Table 1:** Metabolites annotated in *Aralia elata* var. *mandshurica* (Rupr. & Maxim.) J. Wen NADES extracts in the experiment of solvent comparison by reversed-phase ultra-high-performance liquid chromatography–tandem mass spectrometry (RP-UHPLC-ESI-LIT-Orbitrap-MS/MS).

#	t_R_ (min)	*m*/*z* [M-H]^−^ Observed	*m*/*z* [M-H]^−^ Calculated	Elemental Composition [M-H]^−^	MS^2^ Fragmentation Patterns—Product Ions, *m*/*z* (rel. Intensity)	Δm (ppm)	Assignment	Fold Change, log_2_(FC) *
**1**	2.8	487.1089	487.1093	C_20_H_23_O_14_^−^	191.0546 (5), 295.0452 (5), 323.0779 (2), 353.0869 (100), 371.0973 (10), 469.0923 (7)	0.8	Malonylhydroxy-dihydrocaffeoyl-quinic acid	2.9 ↓
**2**	2.9	469.0987	469.0988	C_20_H_21_O_13_^−^	191.0559 (10), 353.0878 (100)	0.3	Malonylcaffeoyl quinic acid	4.3 ↓
**3**	3.8	517.1586	517.1563	C_22_H_29_O_14_^−^	191.0559 (40), 309.0946 (5), 353.0871 (100)	−4.5	Pentofuranosyl-dihydrocaffeoyl quinic acid	2.9 ↑
**4**	4.6	577.1634	577.1622	C_20_H_33_O_19_^−^	181.0716 (12), 261.0612 (9), 279.0716 (100), 297.0822 (33), 377.1450 (2), 443.1400 (10), 461.1507 (27), 559.1511 (5)	2.1	Myo-inositol derivatives	2.9 ↑
**5**	5.5	225.0761	225.0768	C_11_H_13_O_5_^−^	107.0503 (5), 137.0973 (4), 163.0764 (15), 181.0869 (23), 207.0660 (100), 225.0761 (3)	1.0	3-hydroxy-5-(2-methoxy-1-methylethoxy)benzoate	3.6 ↓
**6**	6.1	249.0550	249.0557	C_16_H_9_O_3_^−^	115.0038 (20), 133.0143 (100), 205.0504 (7), 231.0664 (3), 249.0550 (3)	2.8	Malic acid derivative	3.4 ↓
**7**	6.1	705.1675	705.1672	C_32_H_33_O_18_^−^	339.0503 (3), 487.1212 (3), 513.1025 (100)	−0.4	Caffeoylquinic acid dimer	3.9 ↑
**8**	8.9	243.1237	243.1238	C_12_H_19_O_5_^−^	99.0089 (7), 181.1233 (10), 199.1338 (6), 207.1024 (5), 225.1129 (100), 243.1237 (6)	−0.4	Trihydroxy-dodecadienoic acid	3.2 ↓
**9**	12.4	961.4589	961.4569	C_47_H_74_ClO_18_^−^	565.3505 (3), 631.3851 (3), 733.5586 (3), 763.4248 (10), 793.4357 (8), 925.4802 (100)	−2.1	Chikusetsusaponin IV (syn. Araloside A)	5.5 ↑
**10**	12.8	793.4367	793.4380	C_42_H_65_O_14_^−^	437.3418 (6), 455.3522 (65), 483.3470 (98), 551.3730 (63), 569.3835 (45), 631.3836 (20), 731.4355 (22), 793.4367 (100)	1.6	Oleanolic acid hexuronide–hexoside	7.9 ↑
**11**	12.8	763.4286	763.4274	C_41_H_63_O_13_^−^	437.3411 (5), 455.3524 (30), 523.3785 (27), 569.3837 (65), 613.3735 (100), 632.3838 (15), 763.4286 (45)	−1.6	Pseudoginsenoside Rp1	5.5 ↑

* FC (fold change) was calculated as the ratio of the recoveries of the metabolite using NADES1 to that of the recoveries of the metabolite using NADES2; log_2_(FC)—binary logarithm of fold change. As can be seen from Table 1, compounds **3**, **4**, **7**, **9**, **10**, and **11** appeared to be 1.8^2^–2.8^2^ times more abundant in NADES1, whereas compounds **1**, **2**, **5**, **6**, and **8** showed 2.1^2^–2.9^2^-fold higher abundances in NADES2.

**Table 2:** Metabolites annotated in *Aralia elata* var. *mandshurica* (Rupr. & Maxim.) J. Wen NADES extracts in the experiment of extraction method comparison by reversed-phase ultra-high-performance liquid chromatography–tandem mass spectrometry (RP-UHPLC-ESI-LIT-Orbitrap-MS/MS).

No	t_R_ (min)	*m*/*z* [M-H]^−^ Observed	*m*/*z* [M-H]^−^ Calculated	Elemental Composition [M-H]^−^	MS2 Fragmentation Patterns—Product Ions, *m*/*z* (rel. Intensity)	Δm (ppm)	Assignment	Fold Change, log_2_(FC) *
**12**	2.9	469.0987	469.0988	C_20_H_21_O_13_^−^	191.0559 (10), 353.0878 (100)	0.3	5-*O*-malonil-caffeoylquinic acid	4.3 ↓
**13**	9.3	1167.4970	1167.4996	C_54_H_84_ClO_25_^−^	697.3640 (3), 969.4656 (5), 1131.5182 (100)	2.2	3,16-dihydroxyolean-12-en-23,28-dioic acid 28-*O*-6-*O*-3-hydroxy-3-methylglutaryl-hexopyranosyl-hexopyranosyl-hexopyranosyl ester	3.5 ↑
**14**	9.4	1137.4930	1137.4890	C_53_H_82_ClO_24_^−^	823.8554 (3), 939.4536 (5), 975.4535 (5), 1101.5084 (100)	−3.5	Celosin I	3.8 ↑
**15**	9.6	1005.4430	1005.4467	C_48_H_74_ClO_20_^−^	688.4667 (3), 730.2089 (3), 842.4215 (5), 969.4651 (100)	3.7	3-*O*-hexopyranosyl-hexopyranosyl-hexuronopyranosyl melilotigenin isomer 1	3.7 ↑
**16**	9.7	1007.4580	1007.4624	C_48_H_76_ClO_20_^−^	777.4055 (5), 799.3860 (20), 911.4629 (5), 939.4566 (25), 971.4799 (100)	4.4	Sophoraflavoside II isomer 1	3.6 ↑
**17**	9.8	941.4707	941.4752	C_47_H_73_O_19_^−^	465.3363 (3), 537.3576 (15), 583.3629 (100), 627.3525 (35), 669.3629 (15), 733.4151 (5), 777.4045 (40), 819.4150 (10), 941.4707 (5)	4.8	Dihydrogypsogenin 3-*O*-pentopyranosyl-hexpyranoxyl-hexuronopyranoside isomer 1	3.7 ↑
**18**	10.5	955.4914	955.4908	C_48_H_75_O_19_^−^	455.3535 (5), 551.3747 (5), 569.3853 (12), 613.3745 (6), 748.4362 (7), 793.4387 (100), 834.4436 (8), 955.4914 (20)	−0.6	Calendulaglycoside C	5.0 ↑
**19**	11.3	1005.4440	1005.4467	C_48_H_74_ClO_20_^−^	404.2202 (3), 572.4813 (3), 842.4193 (3), 942.4771 (3), 969.4661 (100)	2.7	3-*O*-hexopyranosyl-hexopyranosyl-hexuronopyranosyl melilotigenin isomer 2	4.7 ↑
**20**	11.5	1007.4573	1007.4624	C_48_H_76_ClO_20_^−^	939.4597 (3), 971.4816 (100)	5.0	Sophoraflavoside II isomer 2	3.9 ↑
**21**	11.6	971.4841	971.4857	C_48_H_75_O_20_^−^	407.3312 (15), 471.3464 (10), 567.3672 (15), 747.4294 (10), 790.4078 (30), 809.4285 (45), 925.4777 (7), 971.4841 (100)	1.6	Sophoraflavoside II isomer 3	3.7 ↑
**22**	12.1	941.4711	941.4752	C_54_H_87_O_24_	565.3526 (10), 609.3419 (7), 745.4152 (87), 777.4048 (3), 807.4152 (30), 941. 4695 (100)	4.4	Dihydrogypsogenin 3-O-pentopyranosyl-hexpyranoxyl-hexuronopyranoside isomer 2	3.8 ↑
**23**	12.3	961.4589	961.4569	C_47_H_74_ClO_18_^−^	455.3523 (35), 551.3732 (40), 565.3505 (3), 731.4357 (100), 763.4248 (10), 793.4357 (8), 925.4808 (65)	3.8	Chikusetsusaponin IV (syn. Araloside A)	4.7 ↑
**24**	12.6	845.4110	845.4096	C_42_H_66_ClO_15_^−^	407.3310 (50), 477.3728 (30), 539.3671 (20), 567.3671 (33), 587.3570 (50), 647.3779 (100), 747.4291 (8), 809.4301 (30)	−1.7	Ilexoside XLVIII	3.9 ↑
**25**	12.7	777.4057	777.4067	C_41_H_61_O_14_^−^	469.3318 (3), 565.3526 (3), 583.3625 (15), 627.3519 (100), 645.3624 (20), 777.4057 (3)	1.3	3-*O*-hexopyranosyl-pentopyranosylurs-12,18-diene-24,28-dioic acid	5.3 ↑
**26**	12.8	793.4367	793.4380	C_42_H_65_O_14_^−^	455.3522 (70), 483.3470 (100), 537.3574 (30), 551.3730 (65), 569.3834 (45), 613.3732 (20), 631.3835 (25), 731.4354 (25), 793.4367 (95)	1.6	Oleanolic acid hexoside–hexuronide	3.9 ↑
**27**	12.9	779.4216	779.4223	C_41_H_63_O_14_^−^	455.3525 (3), 523.3778 (10), 585.3778 (35), 629.3673 (100), 647.3779 (20), 779.4216 (3)	0.9	3-*O*-pentopyranosyl-hexuronopyranosyl hederagenin	3.8 ↑

* FC (fold change) was calculated as the ratio of the recovery of metabolite extracted using the VAE method to the recovery of metabolite extracted using the maceration method; log_2_(FC)—binary logarithm of fold change.

## Data Availability

The data presented in this study are available upon request from the corresponding authors.

## Supplementary Materials

The following supporting information can be downloaded at: https://www.mdpi.com/article/10.3390/ph17030355/s1, Supplementary Information S1—Figure S1–S12: Secondary metabolites annotated based on targeted MS/MS fragmentation patterns in *Aralia elata* var. *mandshurica* (Rupr. & Maxim.) J. Wen extracts in the experiment of solvent comparison (sorbitol–malic acid vs. choline chloride–malic acid) by reversed-phase ultra-high-performance liquid chromatography–tandem mass spectrometry (RP-UHPLC-ESI-LIT-Orbitrap-MS/MS) in the negative ion mode; Figure S13–S34: Secondary metabolites annotated based on targeted MS/MS fragmentation patterns in *Aralia elata* var. *mandshurica* (Rupr. & Maxim.) J. Wen extracts in the experiment of extraction method comparison (maceration vs. vibrocavitator) by reversed-phase ultra-high-performance liquid chromatography–tandem mass spectrometry (RP-UHPLC-ESI-LIT-Orbitrap-MS/MS) in the negative ion mode. Supplementary Information S2—Table S1: MSDial parameter settings for analysis of secondary metabolites of *Aralia elata* extracts. Table S2: Validation of processing method MSDial vs. Xcalibur. Table S3: Extraction protocol of *A. elata*. Table S4: Digital matrix for secondary metabolites of *A. elata* analysis. Table S5: The standardization of the method using three aralosides A, B, and C, previously identified for *A. elata* extracts.
