# Supplementary material for: The Effects of Selected Extraction Methods and Natural Deep Eutectic Solvents on the Recovery of Active Principles from Aralia elata var. mandshurica (Rupr. & Maxim.) J. Wen: A Non-Targeted Metabolomics Approach
Source: Pharmaceuticals (Basel). 2024 Mar 9;17(3):355. doi: 10.3390/ph17030355 (PMC10974460; doi:10.3390/ph17030355)
Supplement: Supplementary file 1 [file pharmaceuticals-17-00355-s001.zip › Supplementary Information S1.pdf]

The Effects of Selected Extraction Methods and Natural Deep Eutectic Solvents on the Recovery of Active Principles from *Aralia elata var. mandshurica* (Rupr. & Maxim.) J. Wen: A Non-Targeted Metabolomics Approach

Alyona Kaleta [1], Nadezhda Frolova [2], Anastasia Orlova [2], Alena Soboleva [2], Natalia Osmolovskaya [3], Elena Flisyuk [1], Olga Pozharitskaya [4], Andrej Frolov [2,*] and Alexander Shikov [1,*]

[1]Department of Technology of Pharmaceutical Formulations, St. Petersburg State Chemical Pharmaceutical University, 197376 Saint-Petersburg, Russia

[2]Laboratory of Analytical Biochemistry and Biotechnology, K.A. Timiryazev Institute of Plant Physiology RAS, 127276 Moscow, Russia

[3]Department of Plant physiology and Biochemistry, St. Petersburg State University, Univeritetskaya Nab. 7-9, 199034 St. Petersburg, Russia

[4]Research Group of Biochemistry and Technology of Hydrobionts of Algae and Invertebrates, Murmansk Marine Biological Institute of the Russian Academy of Sciences (MMBI RAS), 17 Vladimirskaya Str., 183010 Murmansk, Russia.

*Correspondence: frolov@ifr.moscow (A.F.); alexs79@mail.ru (A.S.)

Supplementary Information S1

Directory

Figures S1-12. Secondary metabolites annotated by targeted MS/MS fragmentation patterns in *Aralia elata var. mandshurica* (Rupr. & Maxim.) J. Wen extracts in experiment of extractant comparison (sorbitol:malic acid vs choline chloride:malic acid) by reversed phase ultra-high-performance liquid chromatography—tandem mass spectrometry (RP-UHPLC-ESI-LIT-Orbitrap-MS/MS) in the negative ion mode. The MS/MS spectra of individual metabolites are listed as Figure S1 – S12…………………………………………………………...3

Figures S13-S34. Secondary metabolites annotated by targeted MS/MS fragmentation patterns in *Aralia elata var. mandshurica* (Rupr. & Maxim.) J. Wen extracts in the experiment of extraction methods comparison (maceration vs vibrocavitator) by reversed phase ultra-high-performance liquid chromatography—tandem mass spectrometry (RP-UHPLC-ESI-LIT-Orbitrap-MS/MS) in the negative ion mode. The MS/MS spectra of individual metabolites are listed as Figure S13 – S34……………………………………………………………………………..15

Figures S1-S12. Secondary metabolites annotated by targeted MS/MS fragmentation patterns in *Aralia elata var. mandshurica* (Rupr. & Maxim.) J. Wen extracts in experiment of extractant comparison (sorbitol:malic acid vs choline chloride:malic acid) by reversed phase ultra-high-performance liquid chromatography—tandem mass spectrometry (RP-UHPLC-ESI-LIT-Orbitrap-MS/MS) in the negative ion mode. The MS/MS spectra of individual metabolites are listed as Figure S1 – S12.

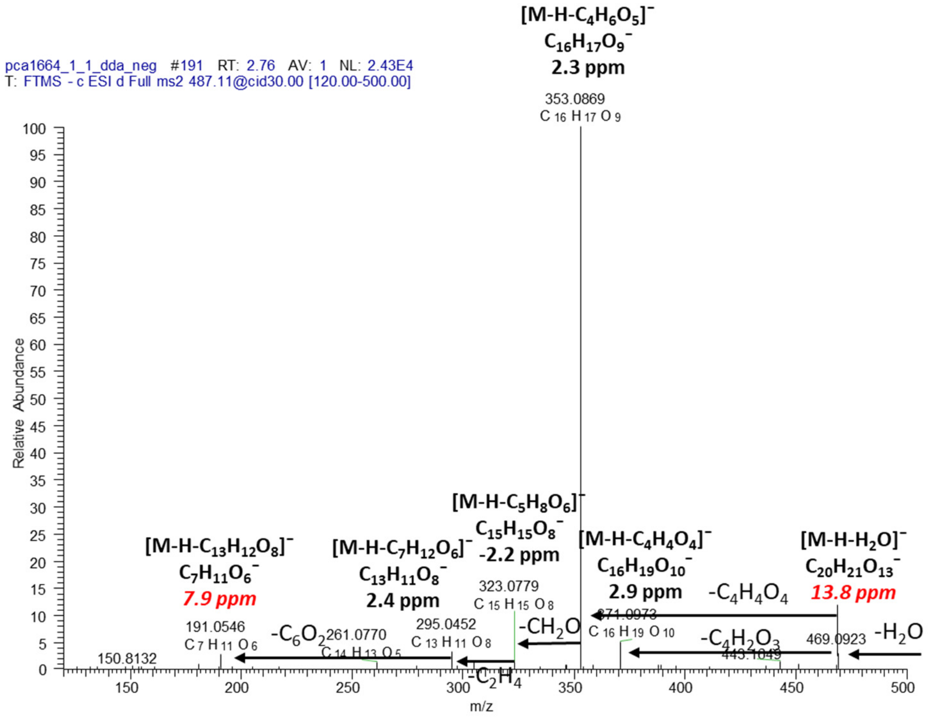

Figure S1. Tandem mass spectrum of *m/z* 487.11 at t_R 2.76 min corresponding to malonyl hydroxydihydrocaffeoylquinic acid (**1**), annotated in *Aralia elata var. mandshurica* (Rupr. & Maxim.) J. Wen extract. The spectrum was acquired with LIT-Orbitrap mass spectrometer operated in the negative product ion mode (collision energy 30 eV).

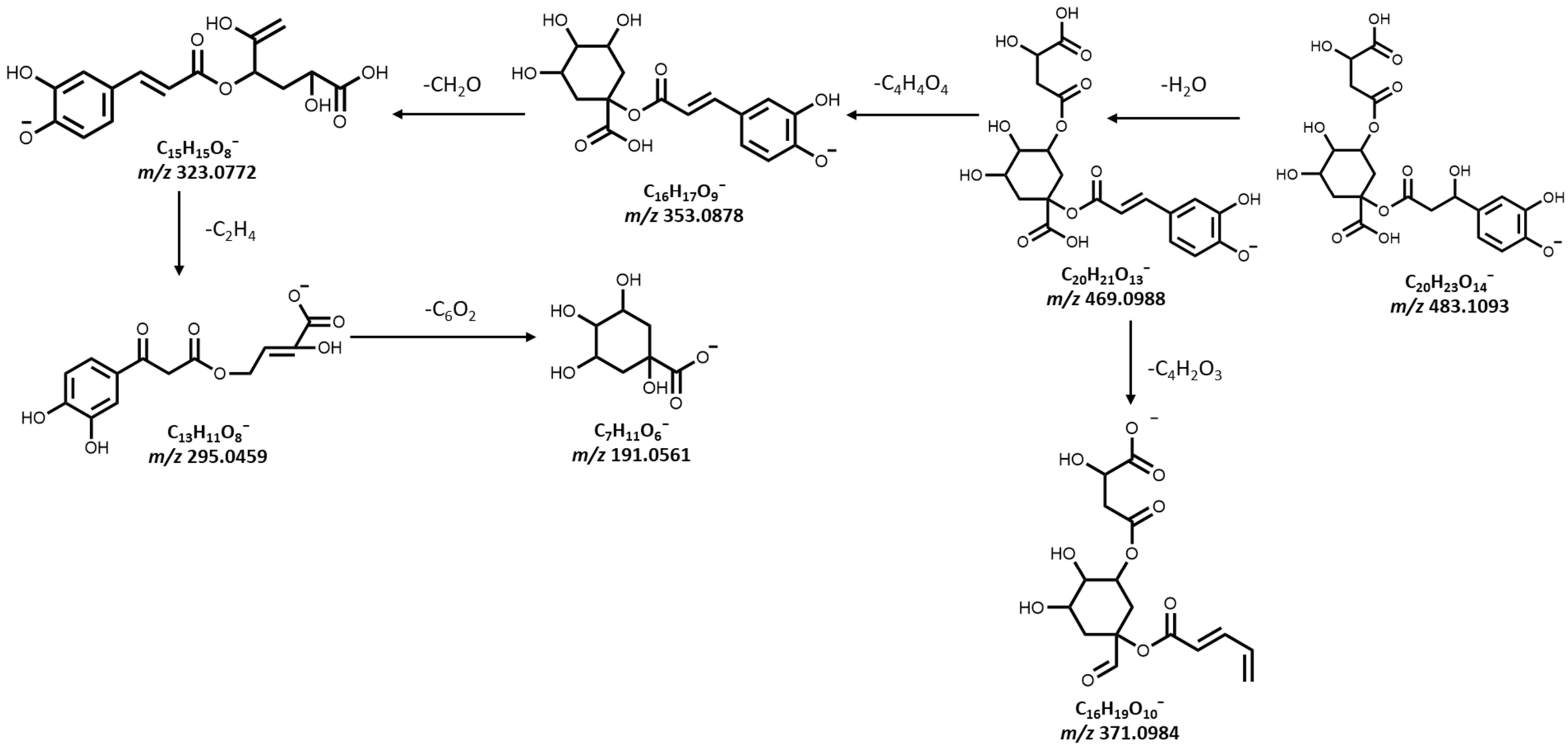

Figure S2. Proposed tandem mass spectrometric fragmentation patterns for the ion at *m/z* 487.11 ($[M-H]^-$, MS^2) corresponding to malonyl hydroxy-dihydrocaffeoylquinic acid (**1**).

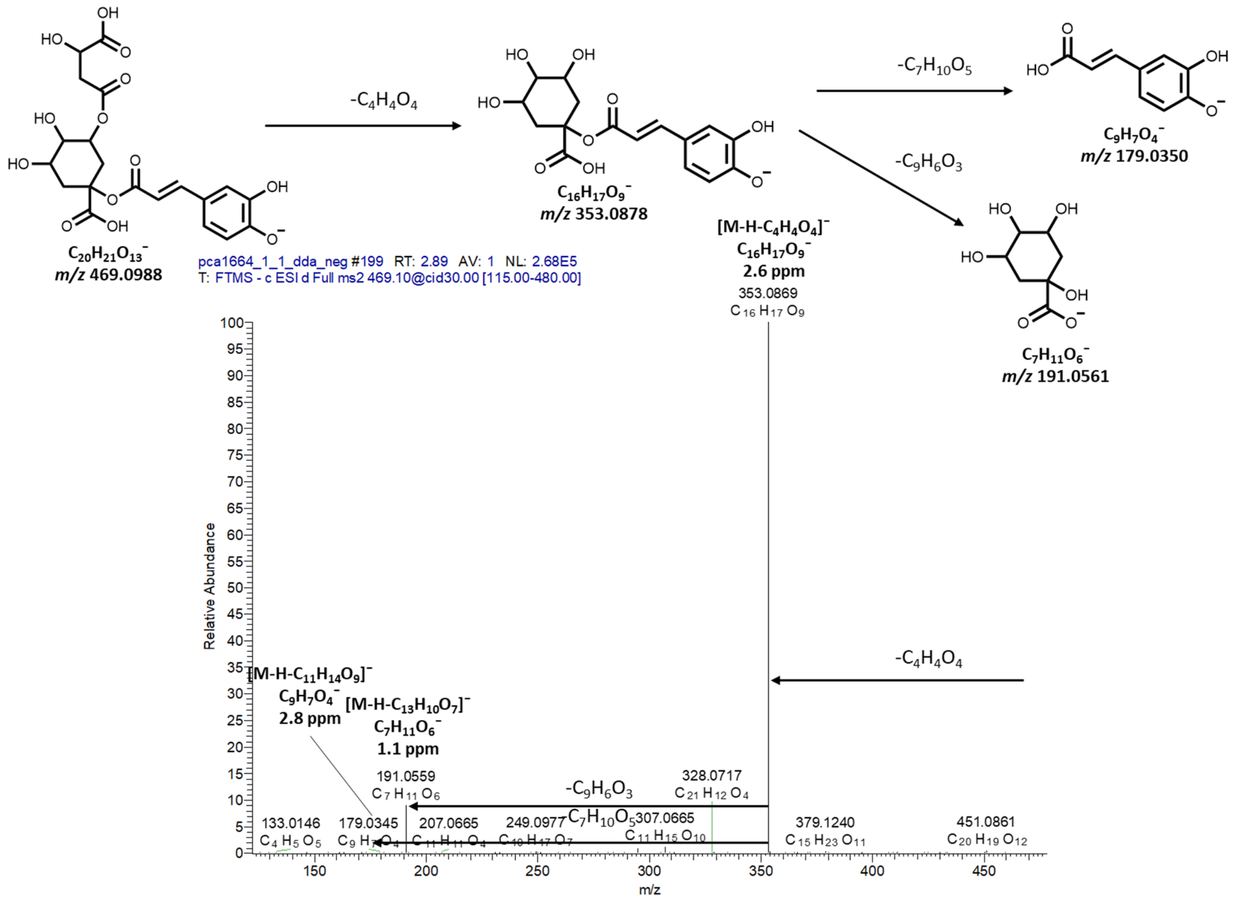

Figure S3. Tandem mass spectrum of *m/z* 469.10 at t_R 2.89 min corresponding to malonyl caffeoylquinic acid (**2**), annotated in *Aralia elata var. mandshurica* (Rupr. & Maxim.) J. Wen extract. The spectrum was acquired with LIT-Orbitrap mass spectrometer operated in the negative product ion mode (collision energy 30 eV). Structures shown are suggestions.

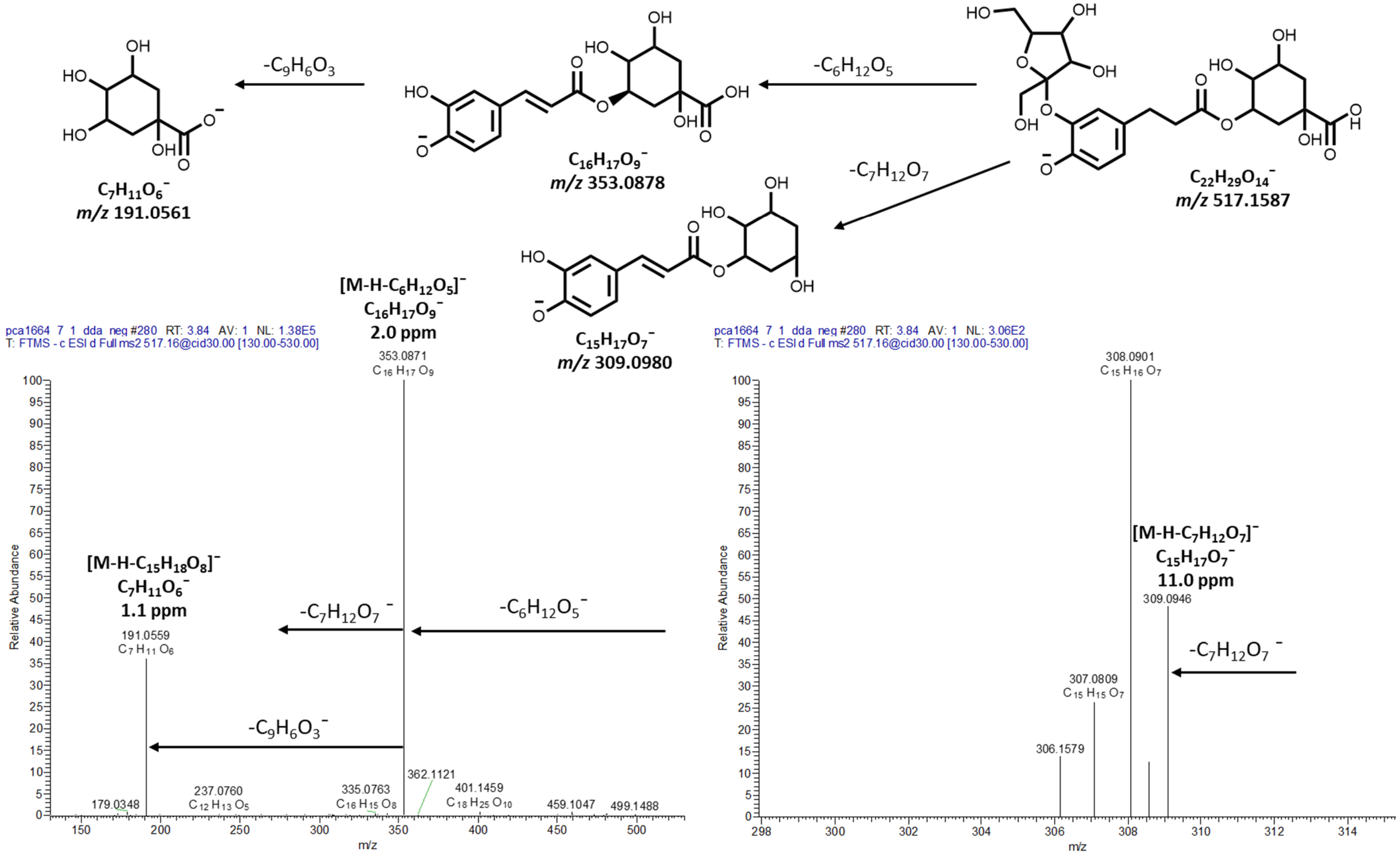

Figure S4. Tandem mass spectrum of *m/z* 517.18 at t_R 3.84 min corresponding to pentofuranosyl dihydrocaffeoylquinic acid (**3**), annotated in *Aralia elata var. mandshurica* (Rupr. & Maxim.) J. Wen extract. The spectrum was acquired with LIT-Orbitrap mass spectrometer operated in the negative product ion mode (collision energy 30 eV). Structures shown are suggestions.

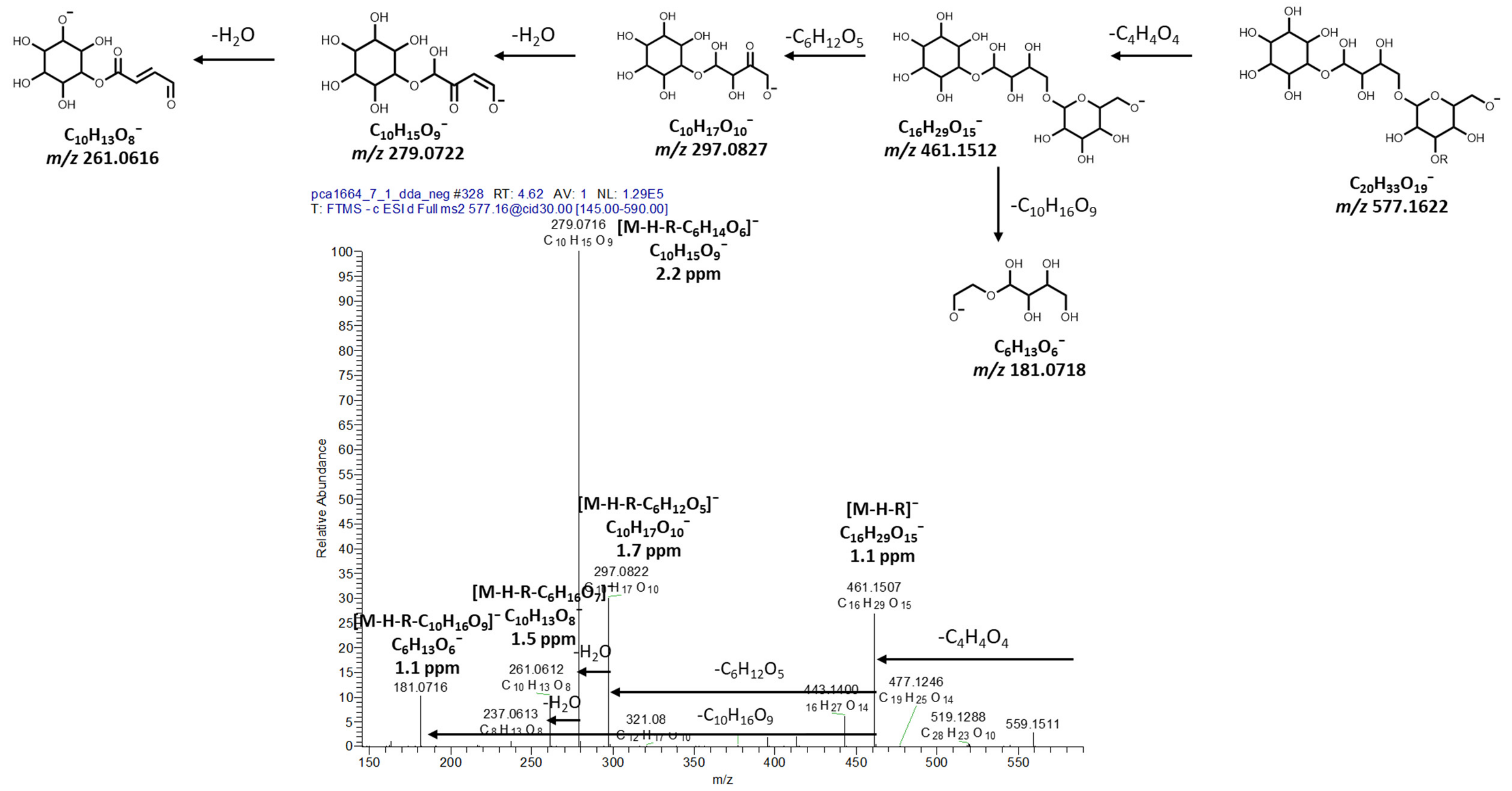

Figure S5. Tandem mass spectrum of *m/z* 577.16 at t_R 4.62 min corresponding to a myo-inositol derivative (**4**), annotated in *Aralia elata var. mandshurica* (Rupr. & Maxim.) J. Wen extract. The spectrum was acquired with LIT-Orbitrap mass spectrometer operated in the negative product ion mode (collision energy 30 eV). Structures shown are suggestions.

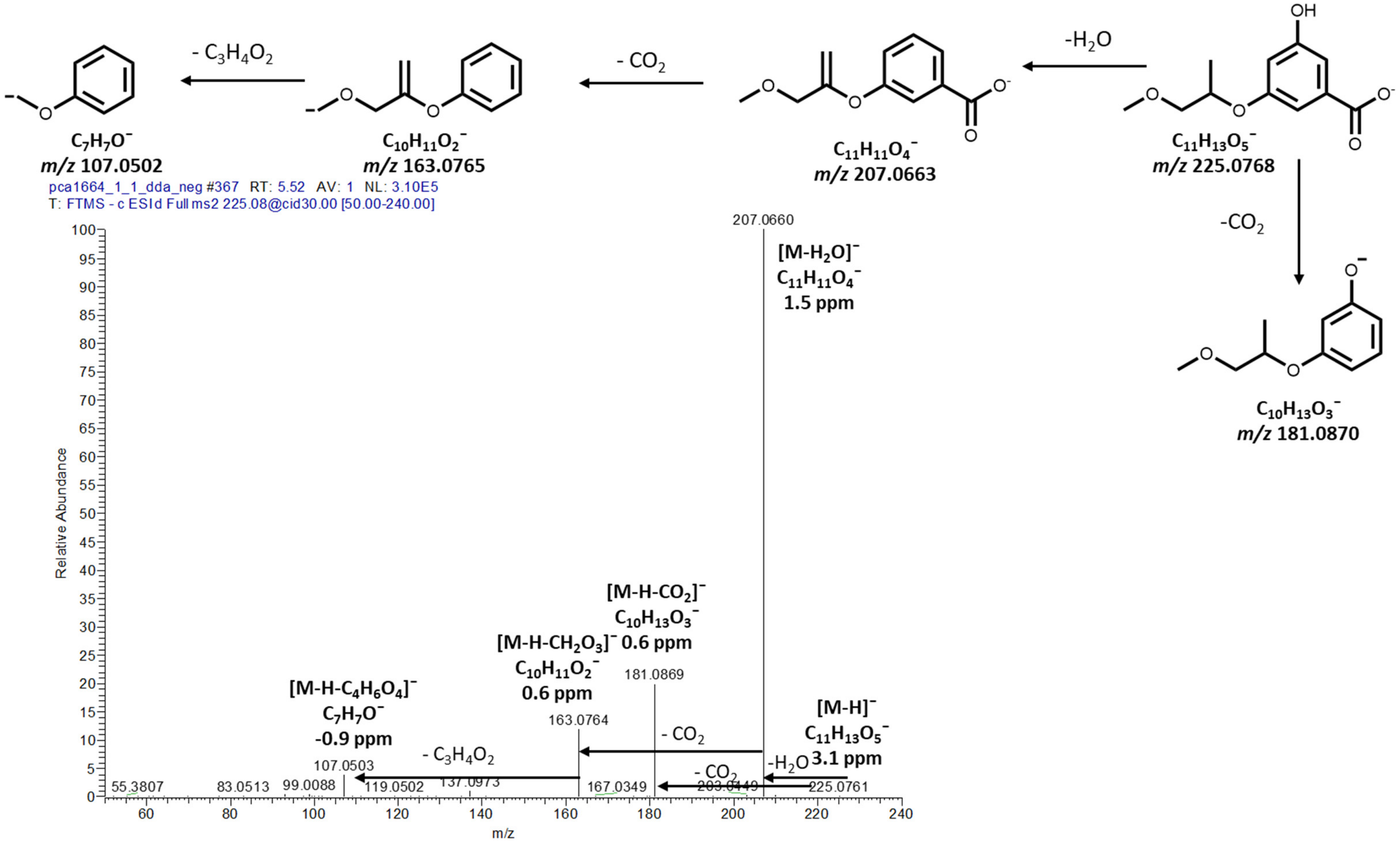

Figure S6. Tandem mass spectrum of *m/z* 225.08 at t_R 5.52 min corresponding to 3-hydroxy-5-(2-methoxy-1-methylethoxy)benzoate (**5**), annotated in *Aralia elata var. mandshurica* (Rupr. & Maxim.) J. Wen extract. The spectrum was acquired with LIT-Orbitrap mass spectrometer operated in the negative product ion mode (collision energy 30 eV). Structures shown are suggestions.

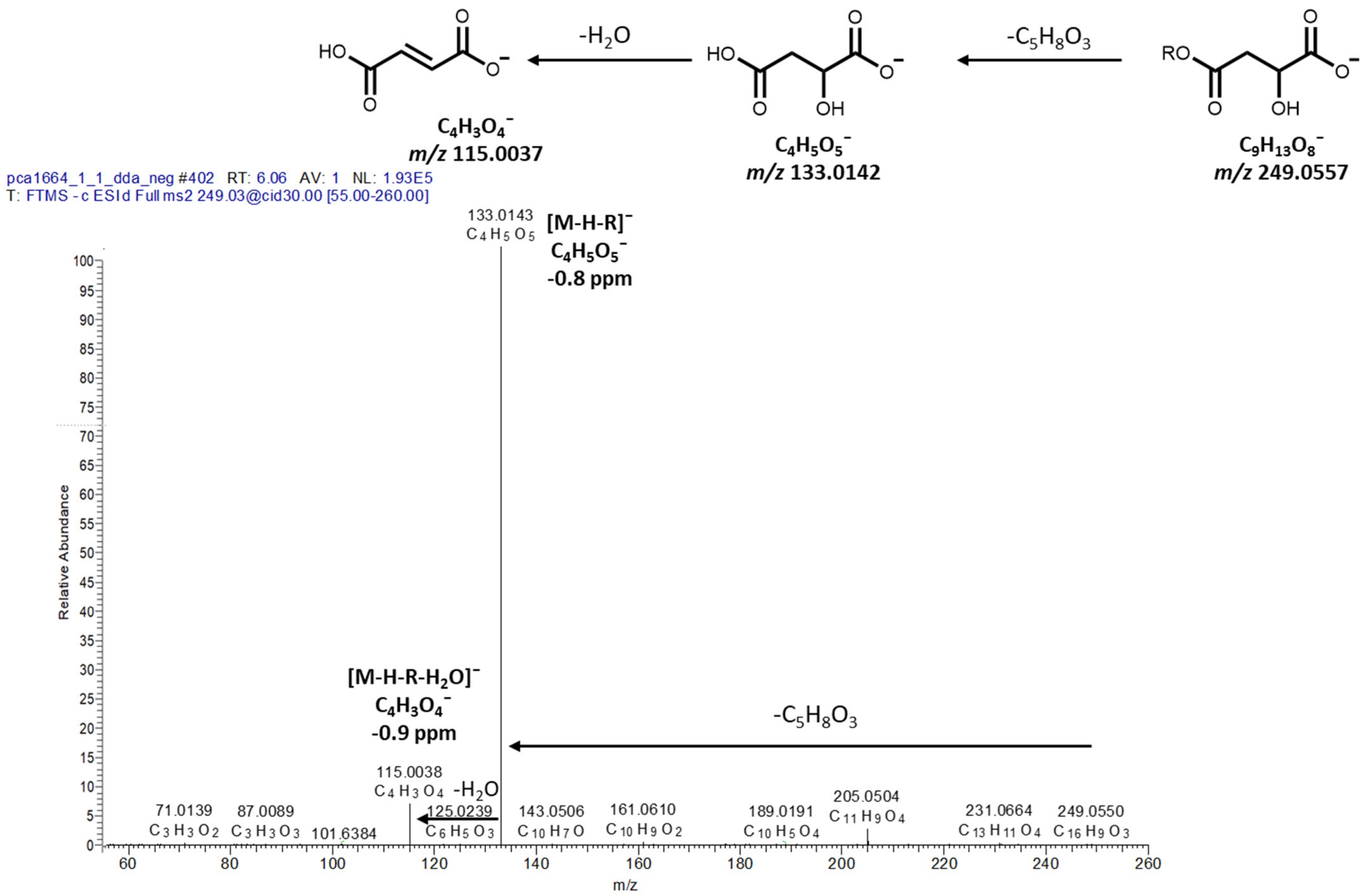

Figure S7. Tandem mass spectrum of *m/z* 249.03 at t_R 6.06 min corresponding to mallic acid derivatives (**6**), annotated in *Aralia elata var. mandshurica* (Rupr. & Maxim.) J. Wen extract. The spectrum was acquired with LIT-Orbitrap mass spectrometer operated in the negative product ion mode (collision energy 30 eV). Structures shown are suggestions.

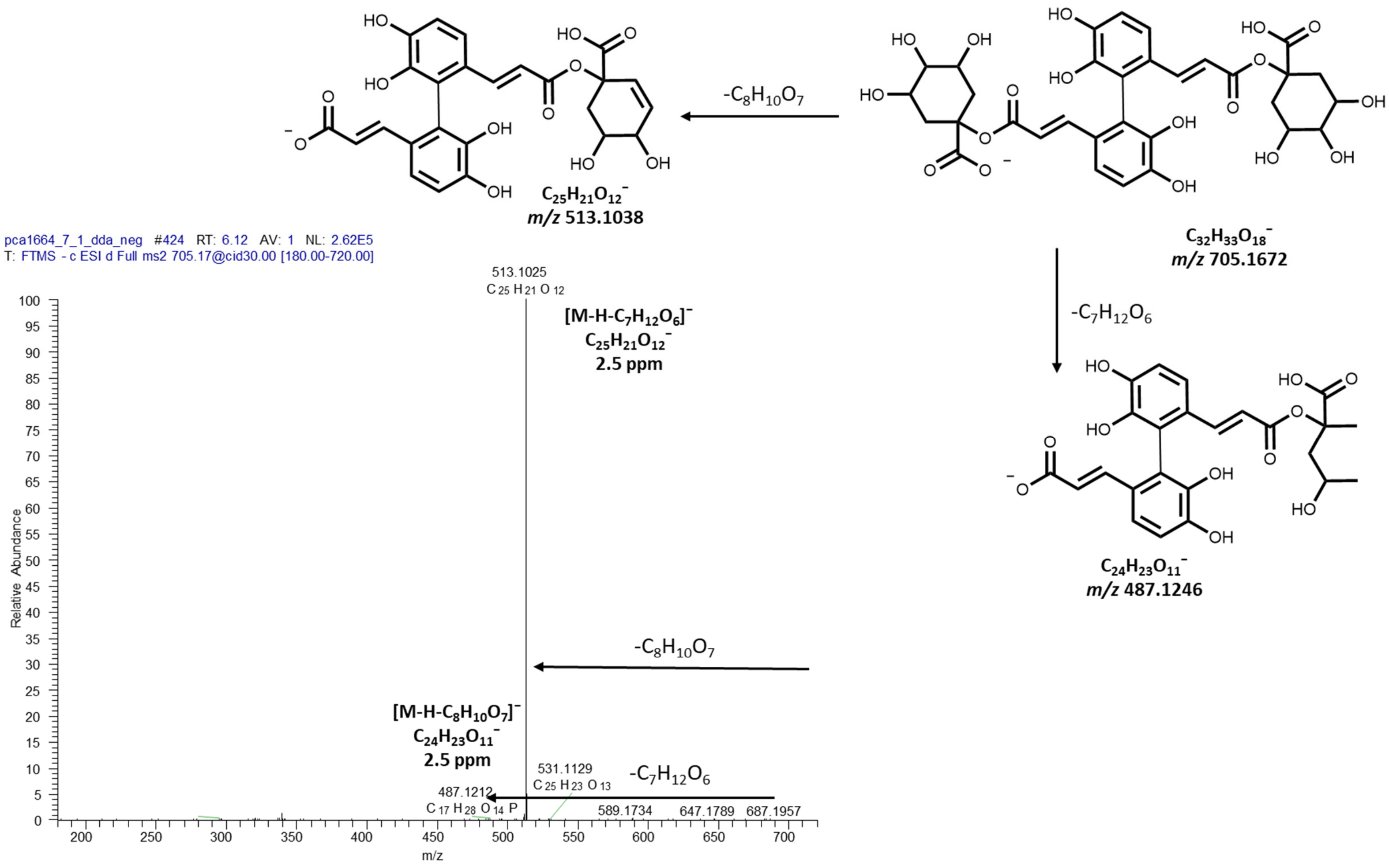

Figure S8. Tandem mass spectrum of *m/z* 705.17 at t_R 6.12 min corresponding to caffeoylquinic acid dimer (**7**), annotated in *Aralia elata var. mandshurica* (Rupr. & Maxim.) J. Wen extract. The spectrum was acquired with LIT-Orbitrap mass spectrometer operated in the negative product ion mode (collision energy 30 eV). Structures shown are suggestions.

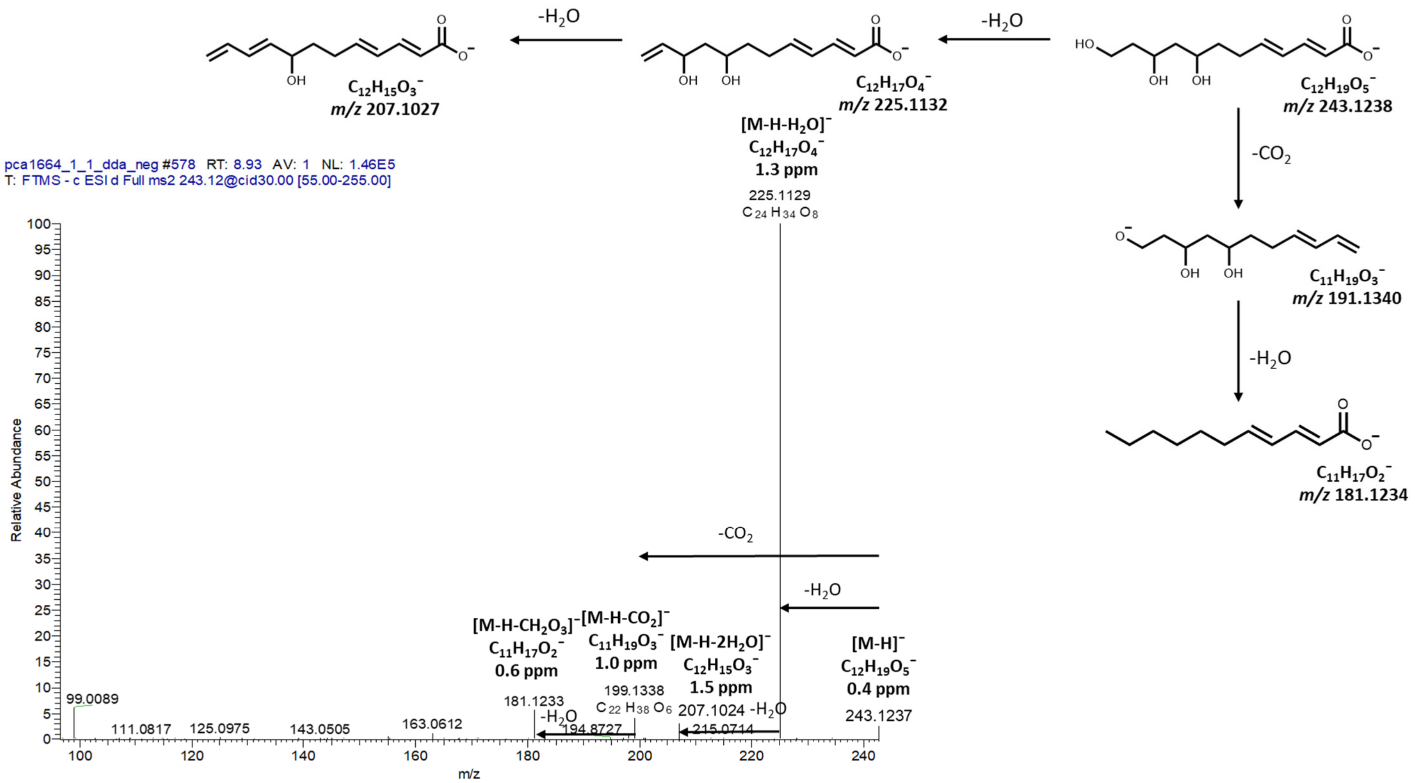

Figure S9. Tandem mass spectrum of *m/z* 243.12 at tR 8.93 min corresponding to trihydroxy-dodecadienoic acid (**8**), annotated in *Aralia elata var. mandshurica* (Rupr. & Maxim.) J. Wen extract. The spectrum was acquired with LIT-Orbitrap mass spectrometer operated in the negative product ion mode (collision energy 30 eV). Structures shown are suggestions.

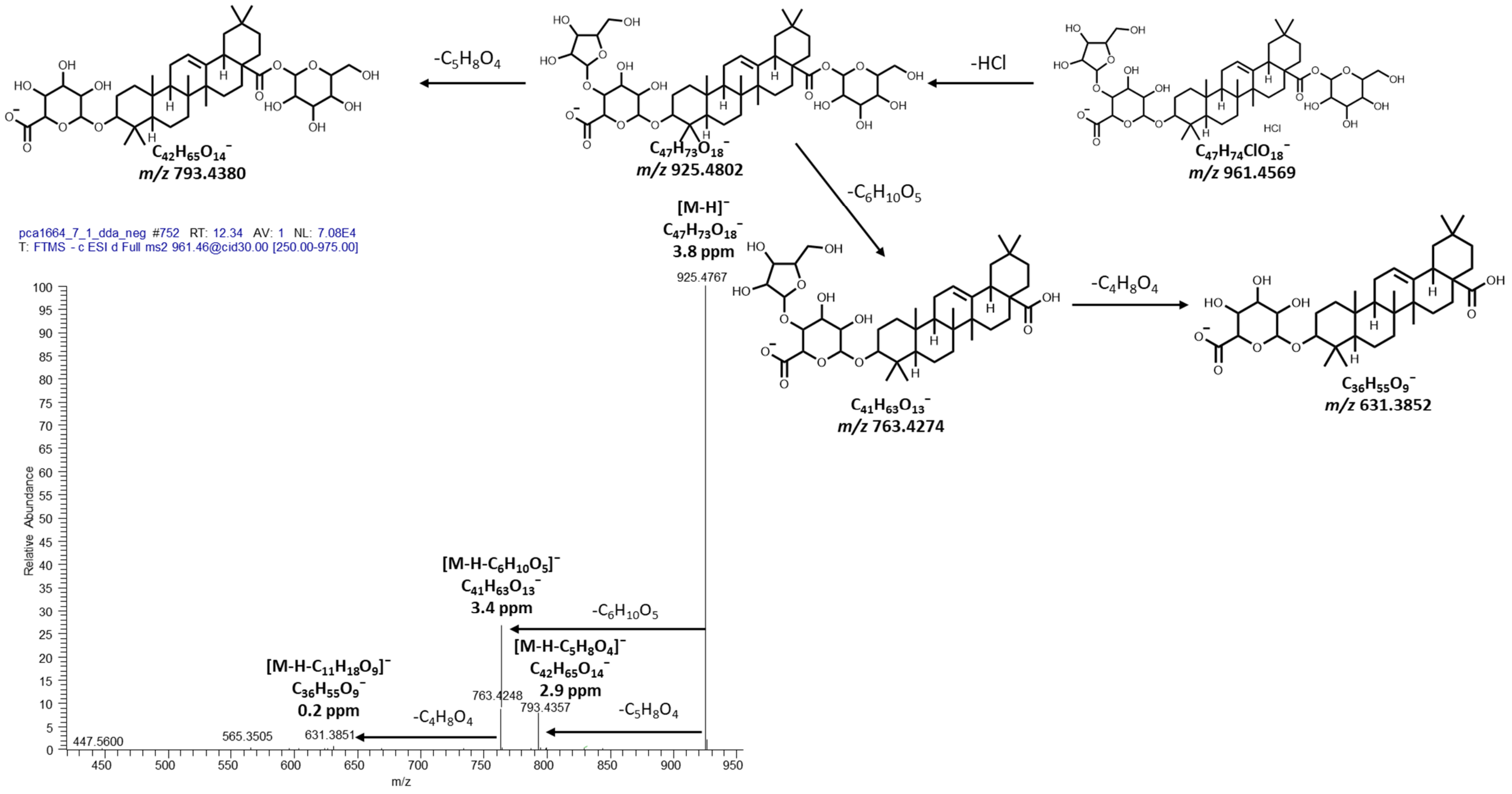

Figure S10. Tandem mass spectrum of *m/z* 961.46 at t_R 12.34 min corresponding to chikusetsusaponin IV (**9**), annotated in *Aralia elata var. mandshurica* (Rupr. & Maxim.) J. Wen extract. The spectrum was acquired with LIT-Orbitrap mass spectrometer operated in the negative product ion mode (collision energy 30 eV). Structures shown are suggestions.

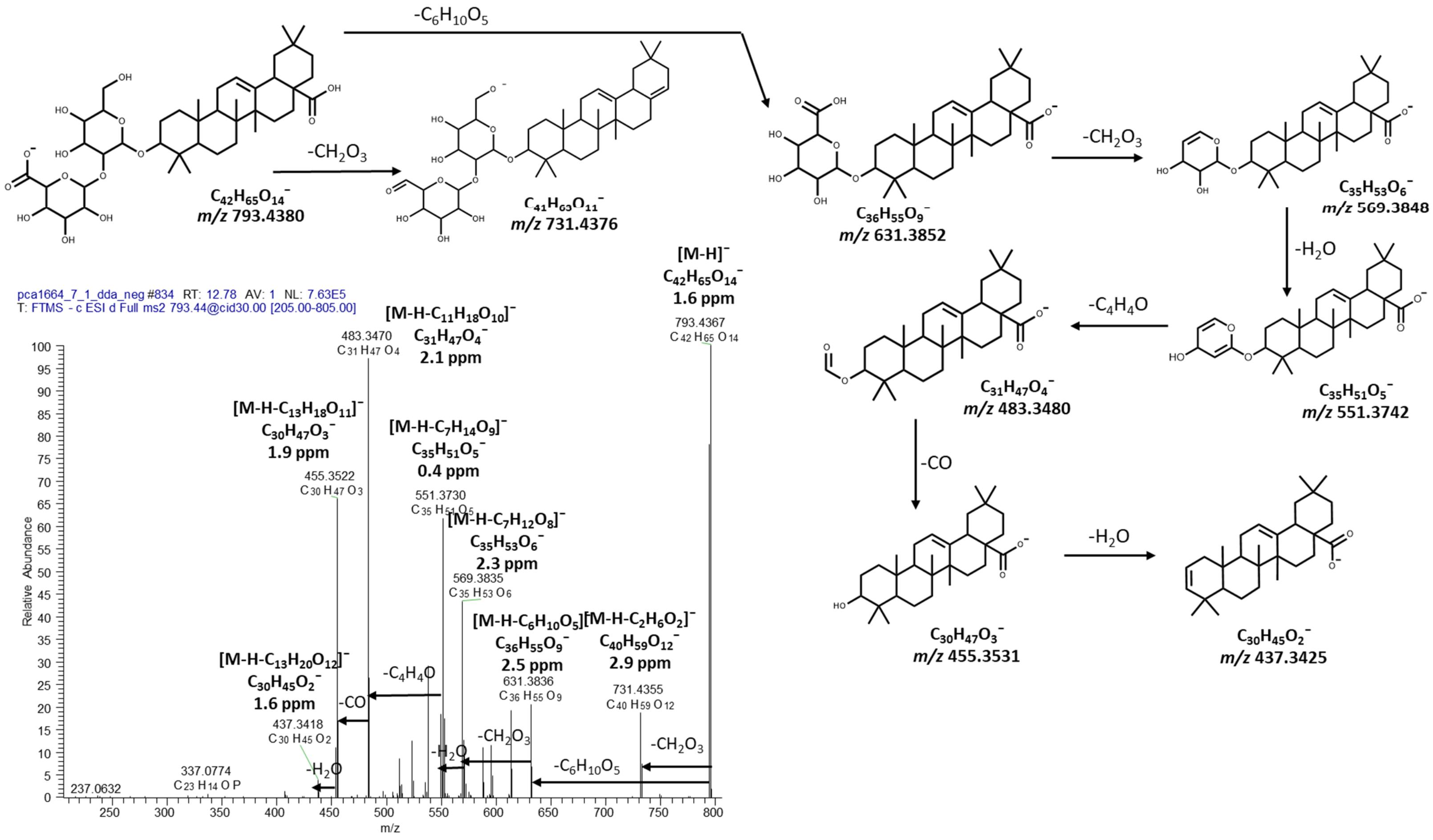

Figure S11. Tandem mass spectrum of *m/z* 793.44 at t_R 12.78 min corresponding to oleanolic acid-hexuronide-hexoside (**10**), annotated in *Aralia elata var. mandshurica* (Rupr. & Maxim.) J. Wen extract. The spectrum was acquired with LIT-Orbitrap mass spectrometer operated in the negative product ion mode (collision energy 30 eV). Structures shown are suggestions.

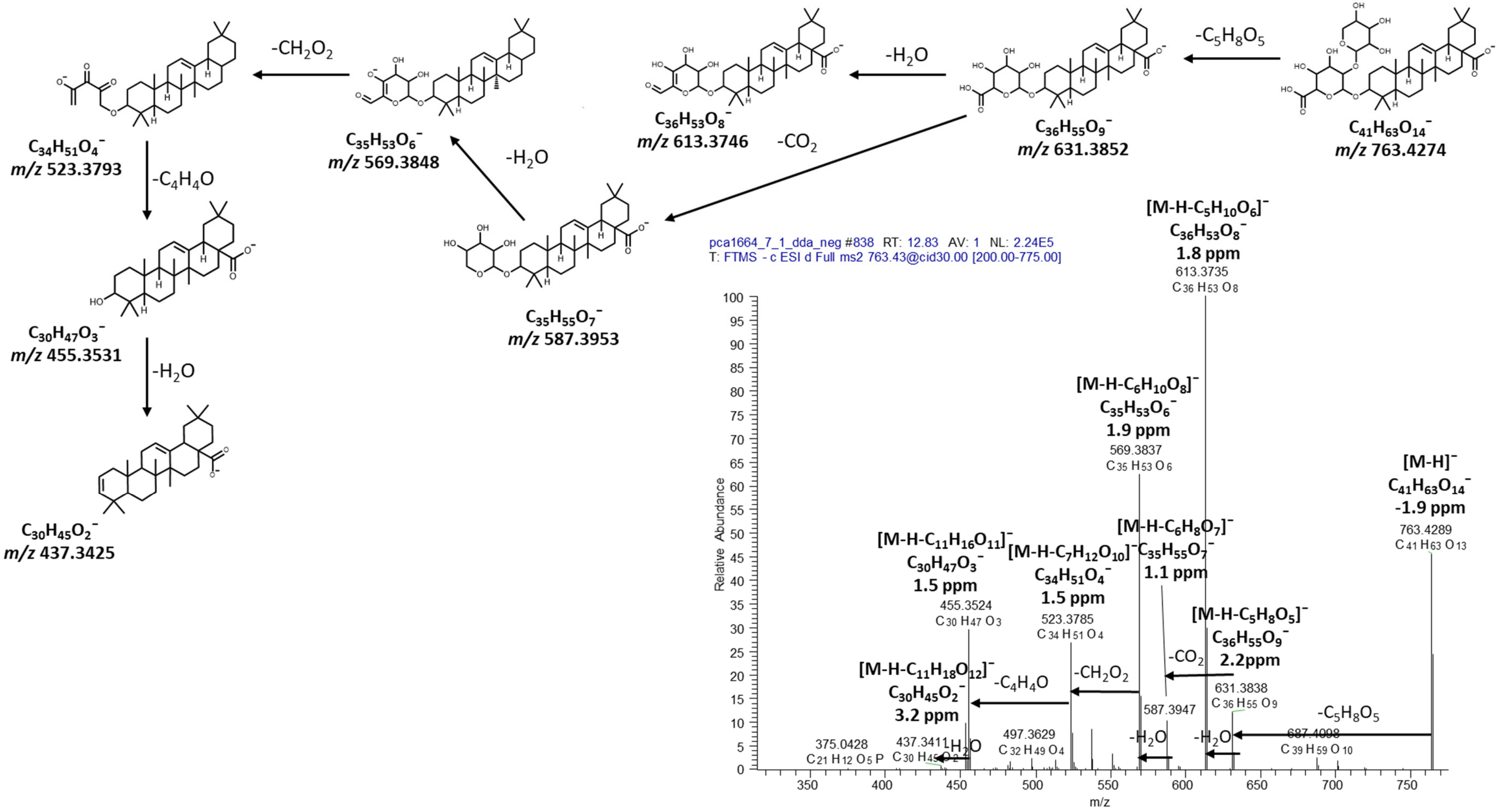

Figure S12. Tandem mass spectrum of *m/z* 763.43 at t_R 12.83 min corresponding to pseudoginsenoside Rp1 (**11**), annotated in *Aralia elata var. mandshurica* (Rupr. & Maxim.) J. Wen extract. The spectrum was acquired with LIT-Orbitrap mass spectrometer operated in the negative product ion mode (collision energy 30 eV). Structures shown are suggestions.

Figures S13 – S34. Secondary metabolites annotated by targeted MS/MS fragmentation patterns in *Aralia elata var. mandshurica* (Rupr. & Maxim.) J. Wen extracts in the experiment of extraction methods comparison (maceration vs vibrocavitator) by reversed phase ultra-high-performance liquid chromatography—tandem mass spectrometry (RP-UHPLC-ESI-LIT-Orbitrap-MS/MS) in the negative ion mode. The MS/MS spectra of individual metabolites are listed as Figure S13 – S34.

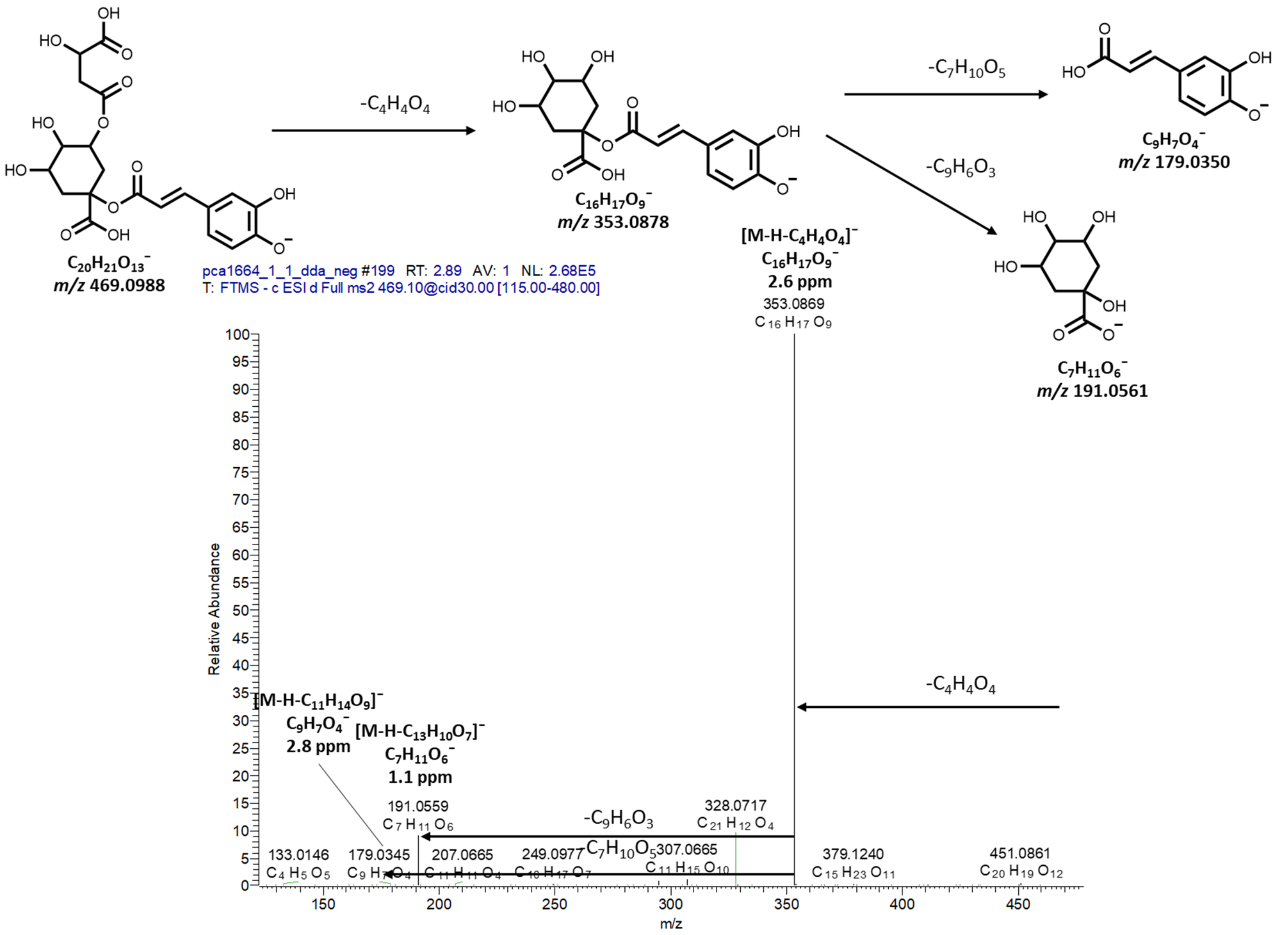

Figure S13. Tandem mass spectrum of *m/z* 469.10 at t_R 2.89 min corresponding to malonyl caffeoylquinic acid (**12**), annotated in *Aralia elata var. mandshurica* (Rupr. & Maxim.) J. Wen extract. The spectrum was acquired with LIT-Orbitrap mass spectrometer operated in the negative product ion mode (collision energy 30 eV). Structures shown are suggestions.

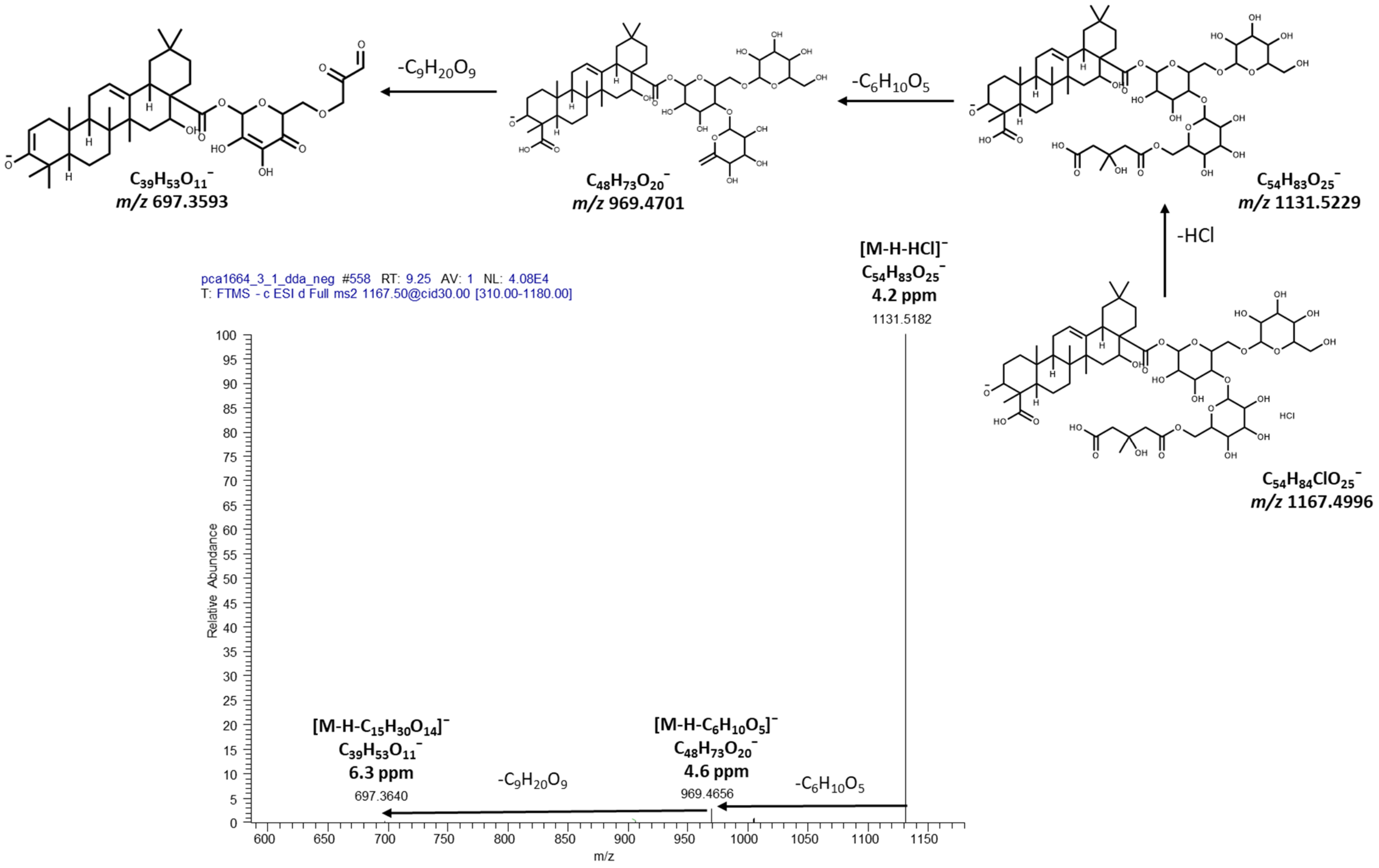

Figure S14. Tandem mass spectrum of *m/z* 1167.50 at tR 9.25 min corresponding to 3,16-dihydroxyolean-12-en-23,28-dioic acid 28-O-[D-6-O-3-hydroxy-3-methylglutar-yl)-hexopyranosyl-(1→3)]-[D-hexopyranosyl-(1→6)]-D-hexopyranosyl ester (**13**), annotated in *Aralia elata var. mandshurica* (Rupr. & Maxim.) J. Wen extract. The spectrum was acquired with LIT-Orbitrap mass spectrometer operated in the negative product ion mode (collision energy 30 eV). Structures shown are suggestions.

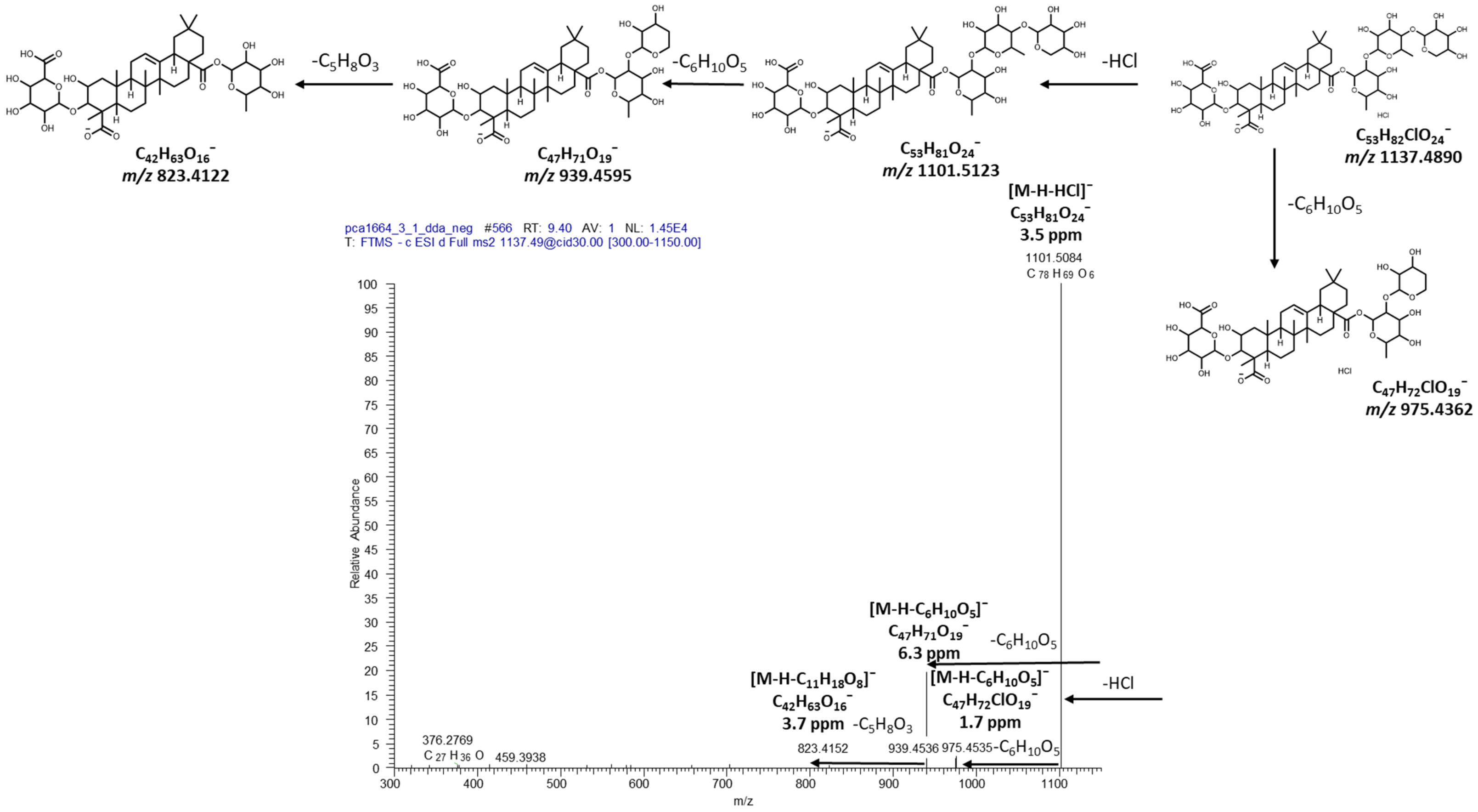

Figure S15. Tandem mass spectrum of *m/z* 1137.49 at t_R 9.40 min corresponding to celosin I (**14**), annotated in *Aralia elata var. mandshurica* (Rupr. & Maxim.) J. Wen extract. The spectrum was acquired with LIT-Orbitrap mass spectrometer operated in the negative product ion mode (collision energy 30 eV). Structures shown are suggestions.

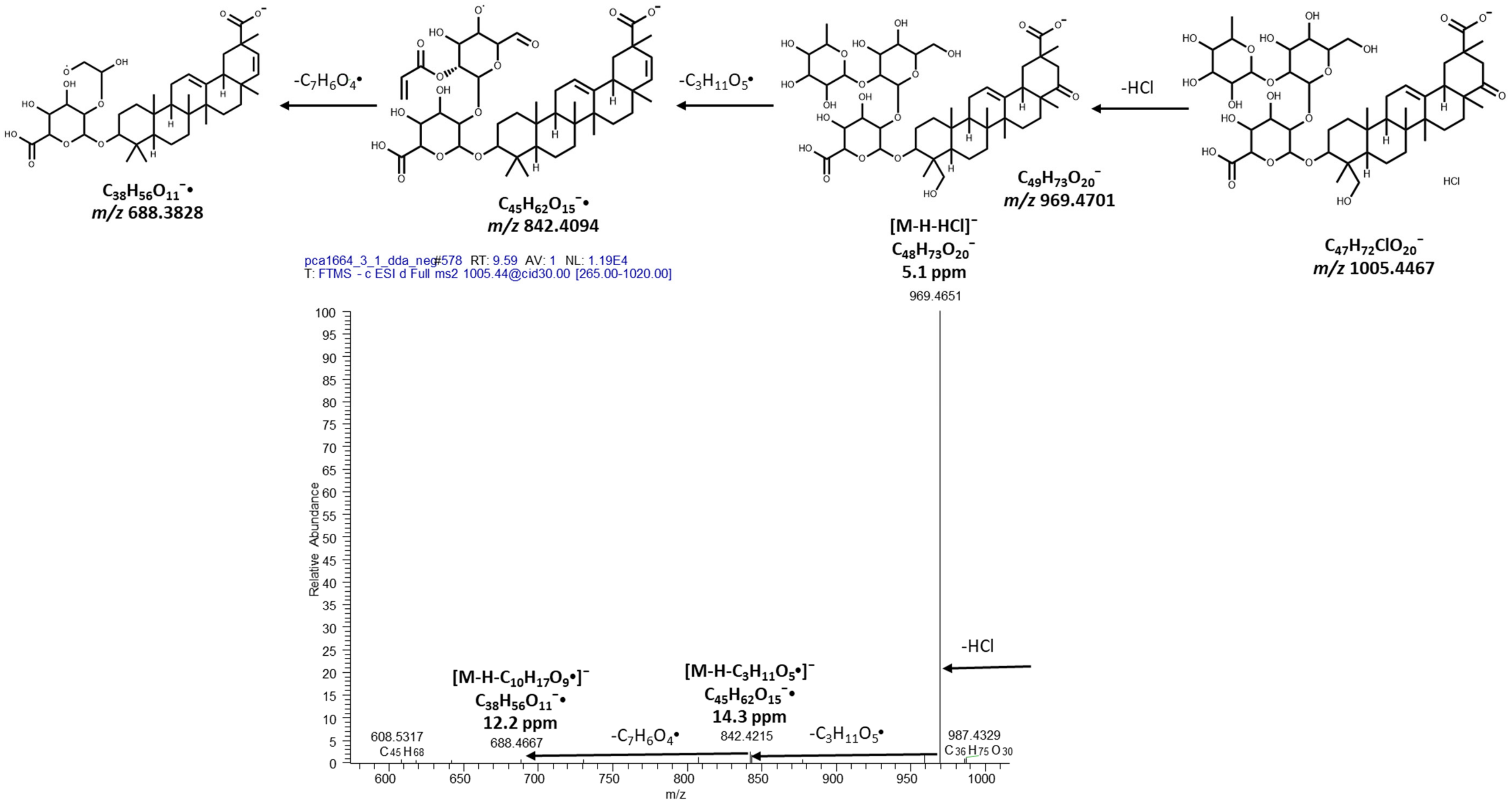

Figure S16. Tandem mass spectrum of *m/z* 1005.44 at t_R 9.59 min corresponding to 3-O-L-hexopyranosyl-(1->2)-D-hexopyranosyl-(1->2)-D-hexuronopyranosyl melilotigenin isomer 1 (**15**), annotated in *Aralia elata var. mandshurica* (Rupr. & Maxim.) J. Wen extract. The spectrum was acquired with LIT-Orbitrap mass spectrometer operated in the negative product ion mode (collision energy 30 eV). Structures shown are suggestions.

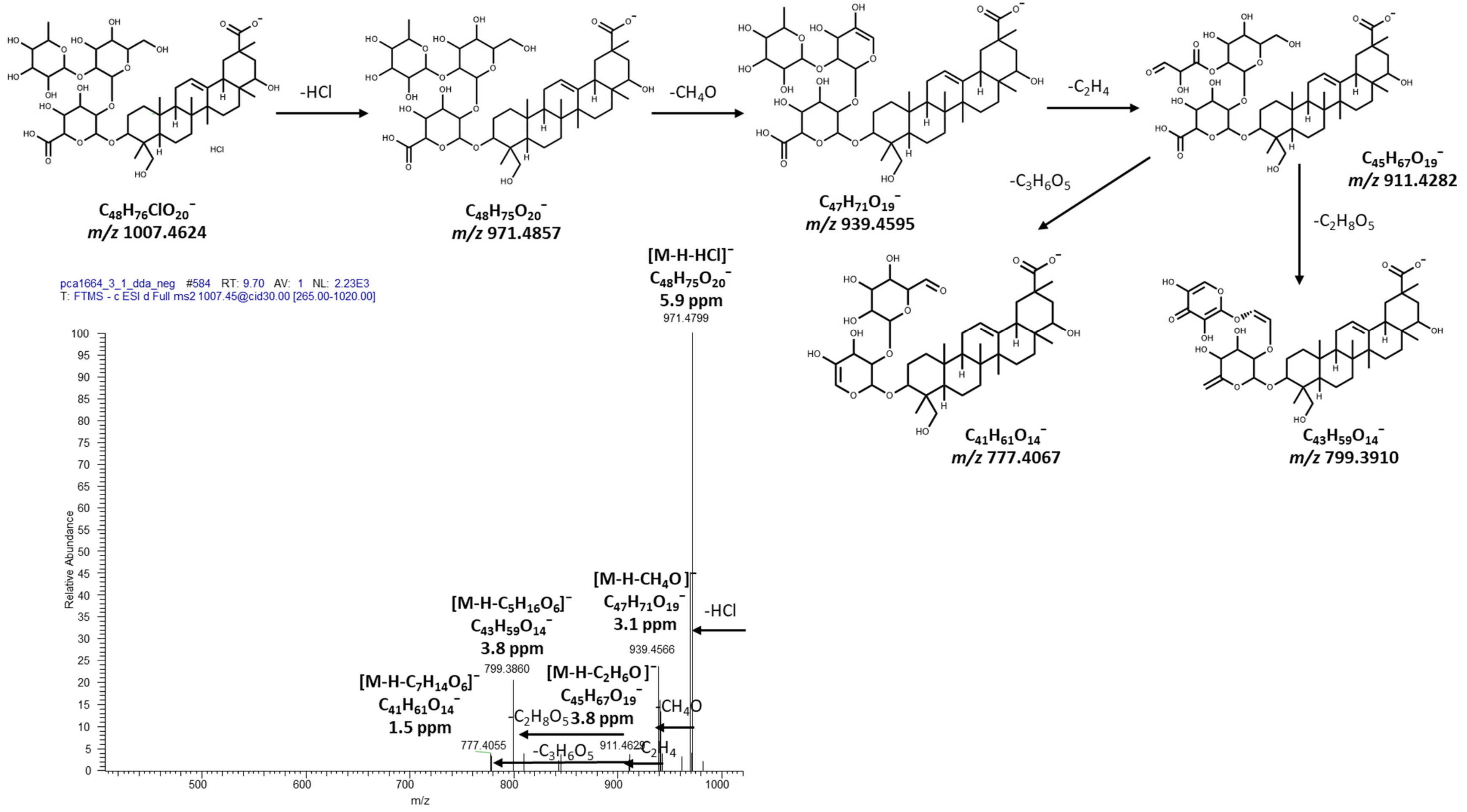

Figure S17. Tandem mass spectrum of *m/z* 1007.45 at t_R 9.70 min corresponding to sophoraflavoside II isomer 1 (**16**), annotated in *Aralia elata var. mandshurica* (Rupr. & Maxim.) J. Wen extract. The spectrum was acquired with LIT-Orbitrap mass spectrometer operated in the negative product ion mode (collision energy 30 eV). Structures shown are suggestions.

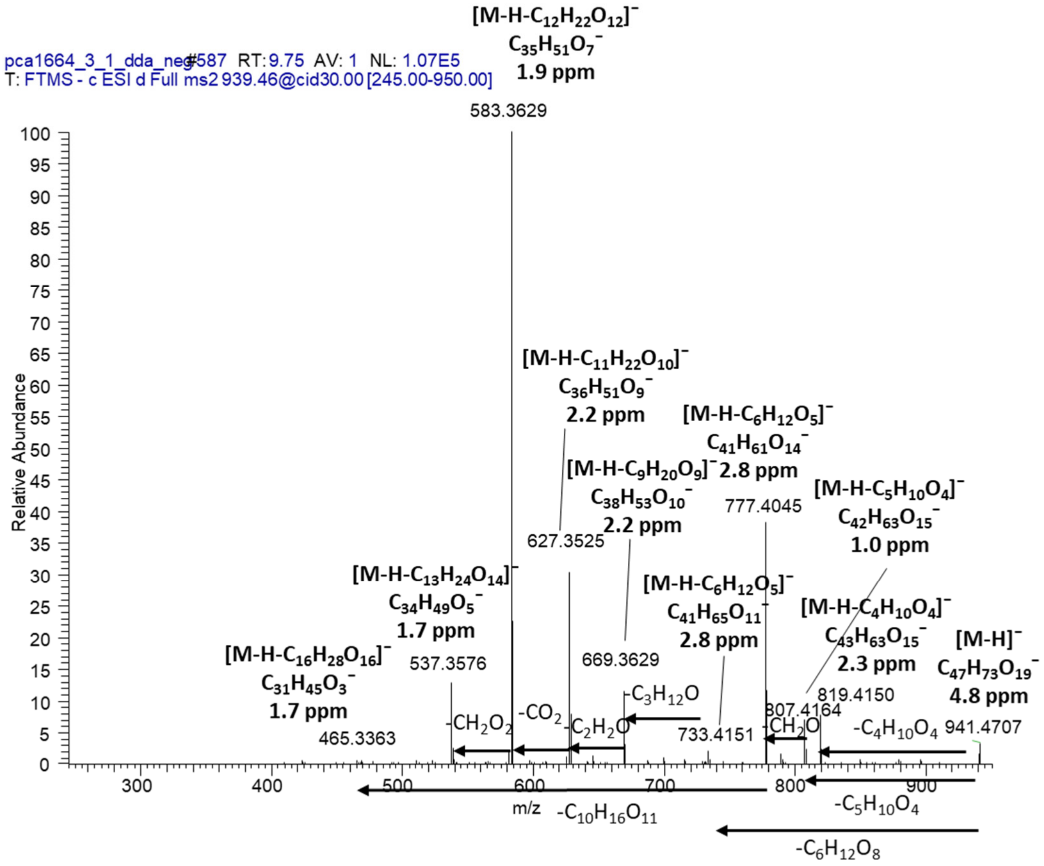

Figure S18. Tandem mass spectrum of *m/z* 939.46 at t_R 9.75 min corresponding to dihydrogypsogenin 3-*O*-pentopyranosyl-(1-2)-hexpyranox-yl-(1-3)>-β-D-hexuronopyranoside isomer 1 (**17**), annotated in *Aralia elata var. mandshurica* (Rupr. & Maxim.) J. Wen extract. The spectrum was acquired with LIT-Orbitrap mass spectrometer operated in the negative product ion mode (collision energy 30 eV).

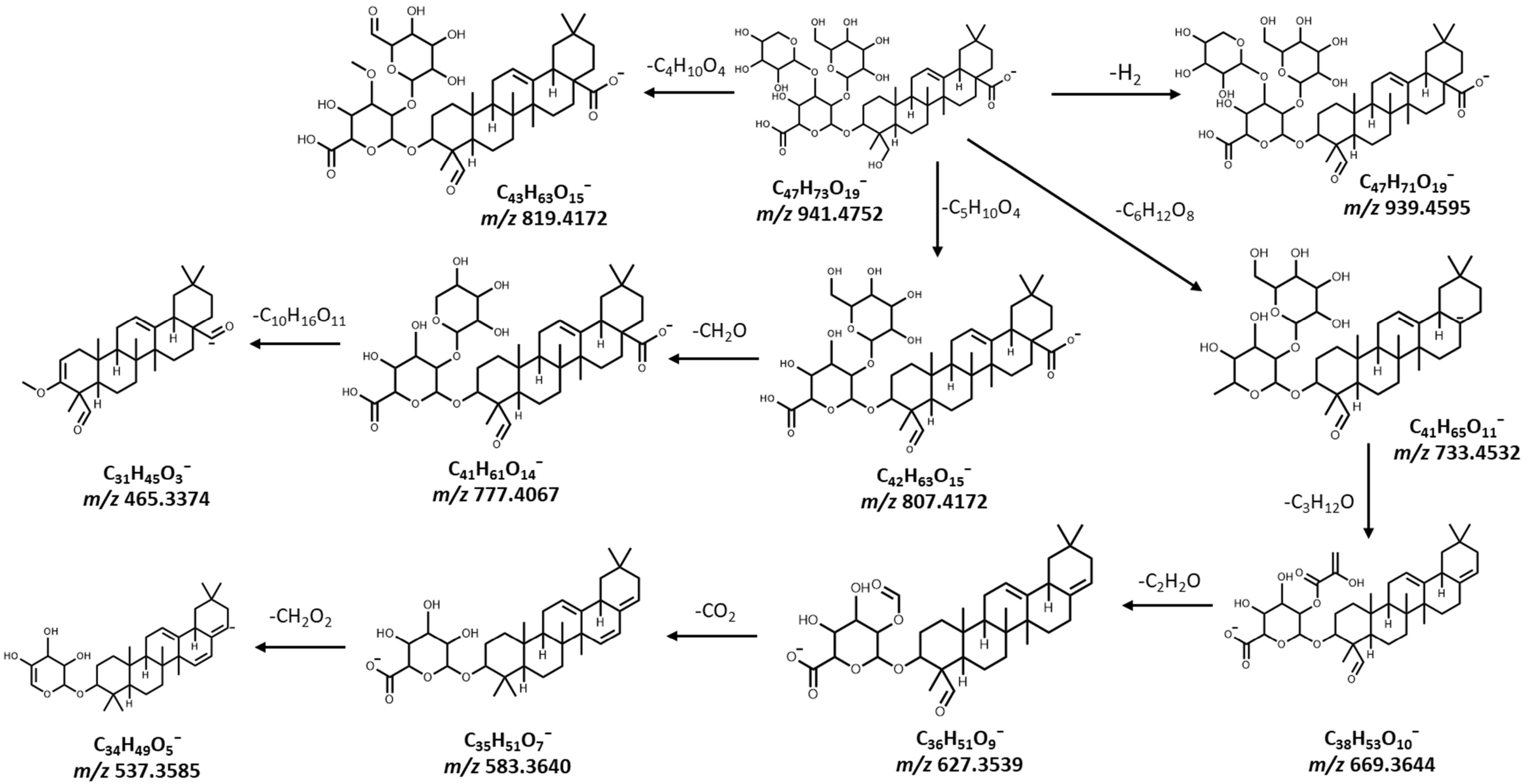

Figure S19. Proposed tandem mass spectrometric fragmentation patterns for the ion at *m/z* 939.46 ([M-H]$^-$, MS^2) corresponding to dihydrogypsogenin 3-O-pentopyranosyl-(1-2)-hexpyranox-yl-(1-3)>-β-D-hexuronopyranoside isomer 1 (**17**).

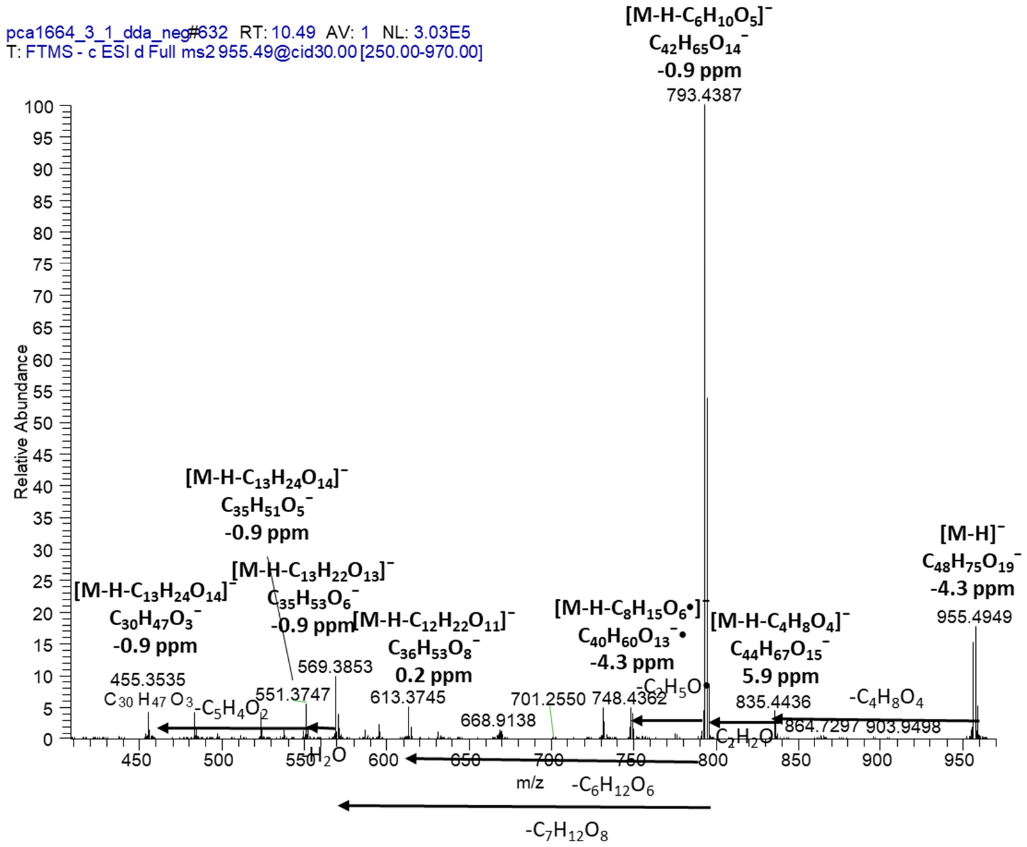

Figure S20. Tandem mass spectrum of *m/z* 955.49 at t_R 10.49 min corresponding to calendulaglycoside C (**18**), annotated in *Aralia elata var. mandshurica* (Rupr. & Maxim.) J. Wen extract. The spectrum was acquired with LIT-Orbitrap mass spectrometer operated in the negative product ion mode (collision energy 30 eV).

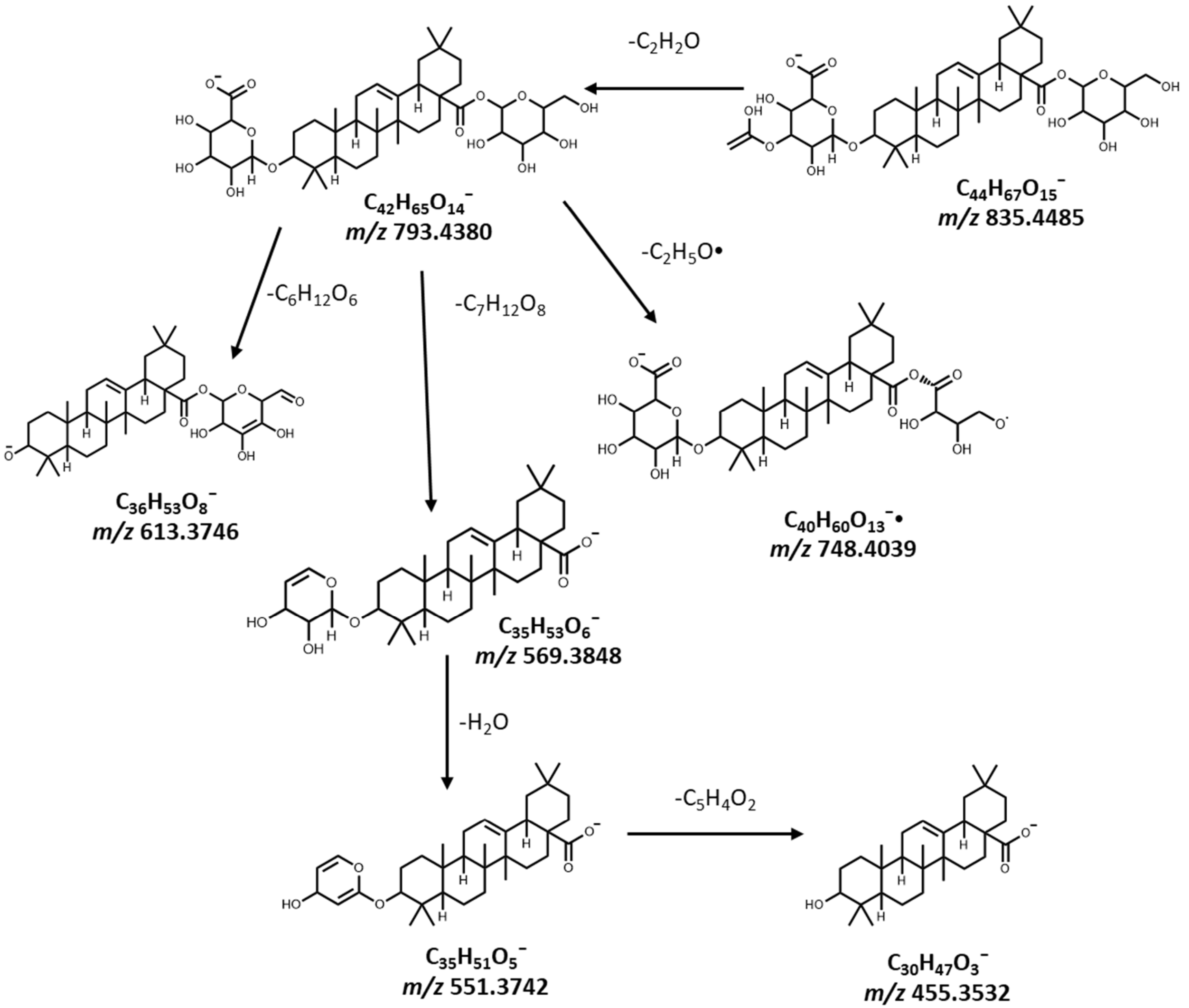

Figure S21. Proposed tandem mass spectrometric fragmentation patterns for the ion at *m/z* 955.49 ($[M-H]^-$, MS^2) corresponding to calendulaglycoside C (**18**).

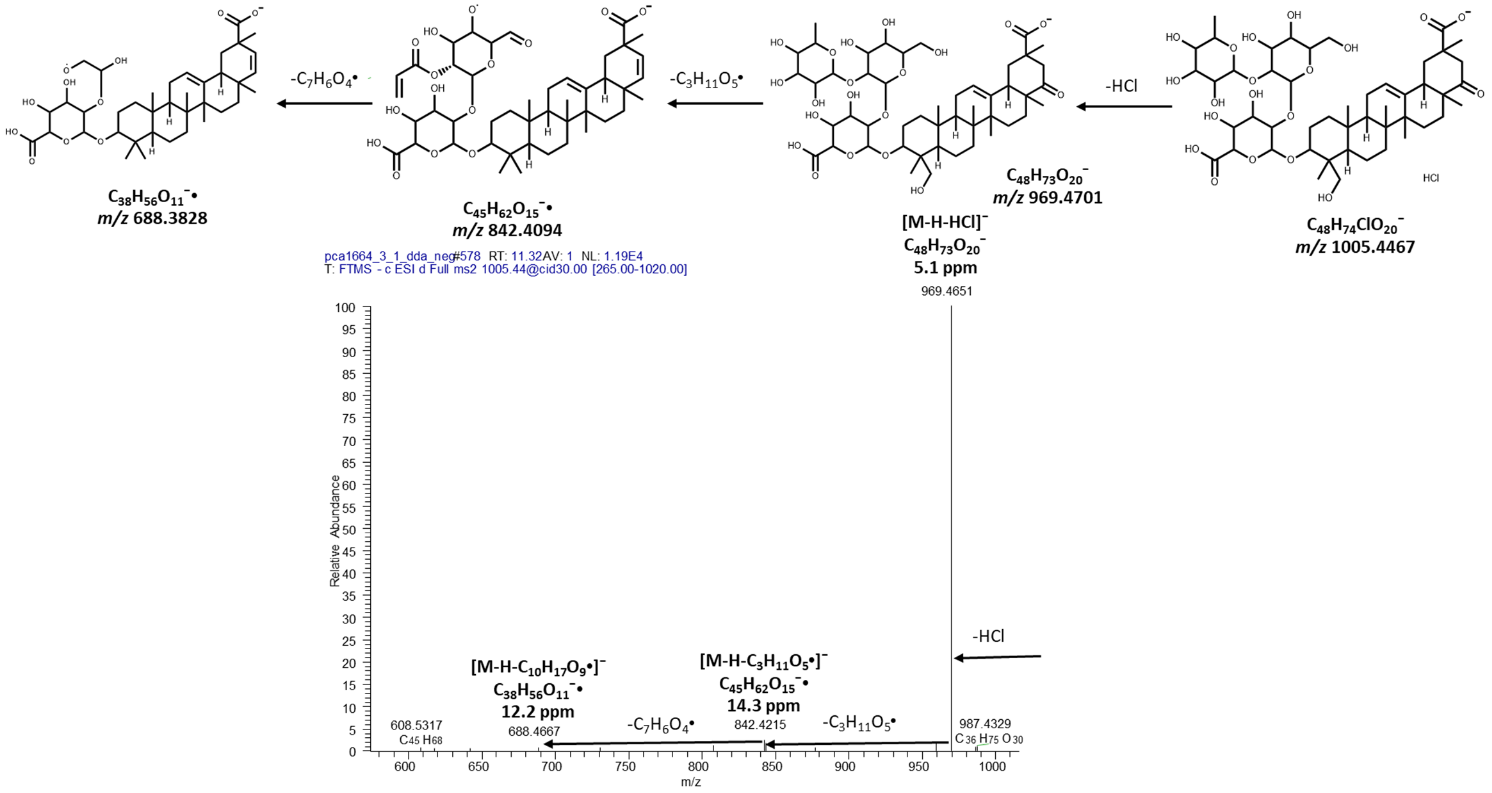

Figure S22. Tandem mass spectrum of *m/z* 955.49 at t_R 10.49 min corresponding to 3-*O*-hexopyranosyl-hexopyranosyl-hexuronopyranosyl melilotigenin isomer 2 (**19**), annotated in *Aralia elata var. mandshurica* (Rupr. & Maxim.) J. Wen extract. The spectrum was acquired with LIT-Orbitrap mass spectrometer operated in the negative product ion mode (collision energy 30 eV). Structures shown are suggestions.

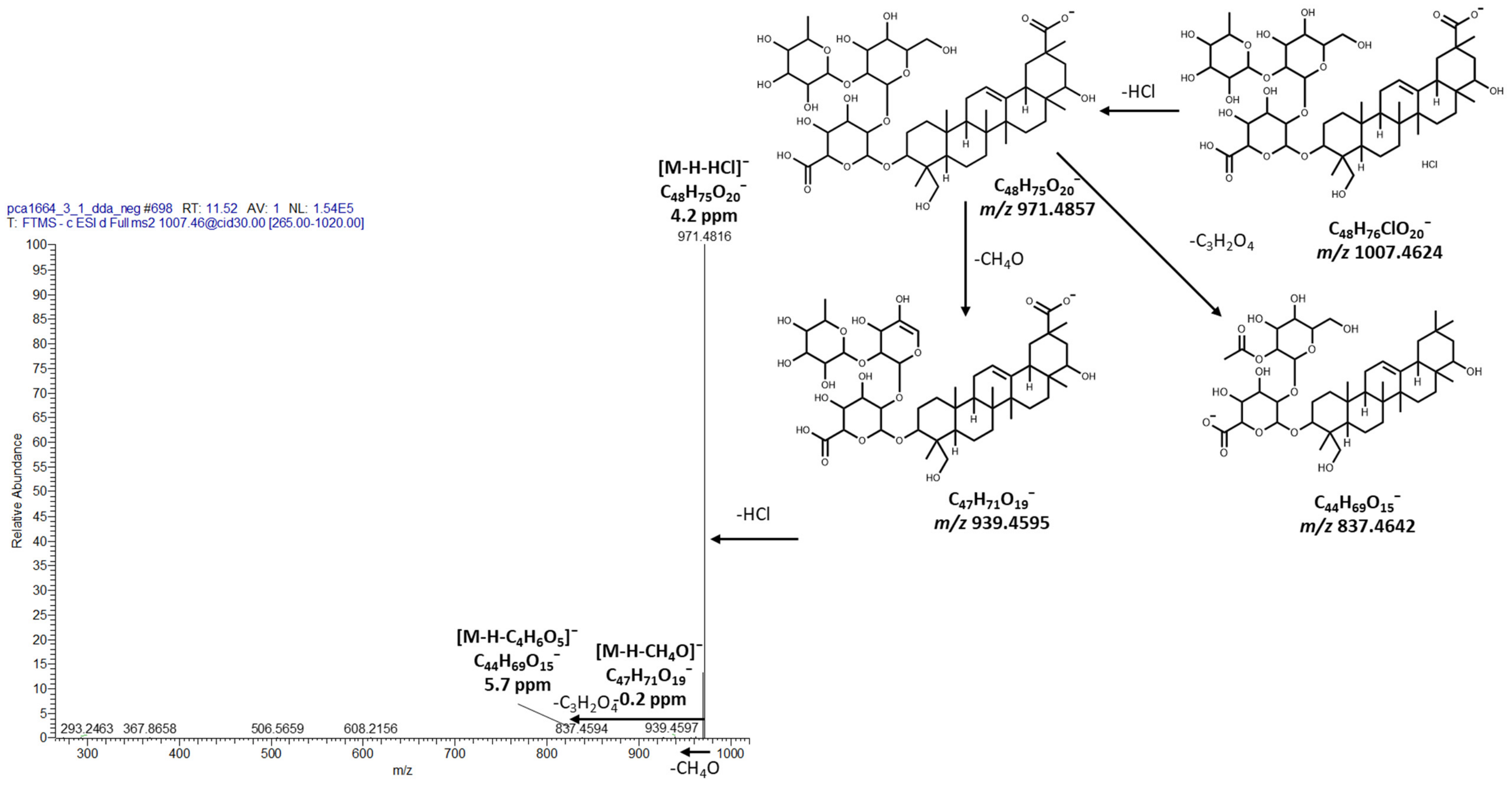

Figure S23. Tandem mass spectrum of *m/z* 1007.46 at t_R 11.52 min corresponding to sophoraflavoside II isomer 2 (**20**), annotated in *Aralia elata var. mandshurica* (Rupr. & Maxim.) J. Wen extract. The spectrum was acquired with LIT-Orbitrap mass spectrometer operated in the negative product ion mode (collision energy 30 eV). Structures shown are suggestions.

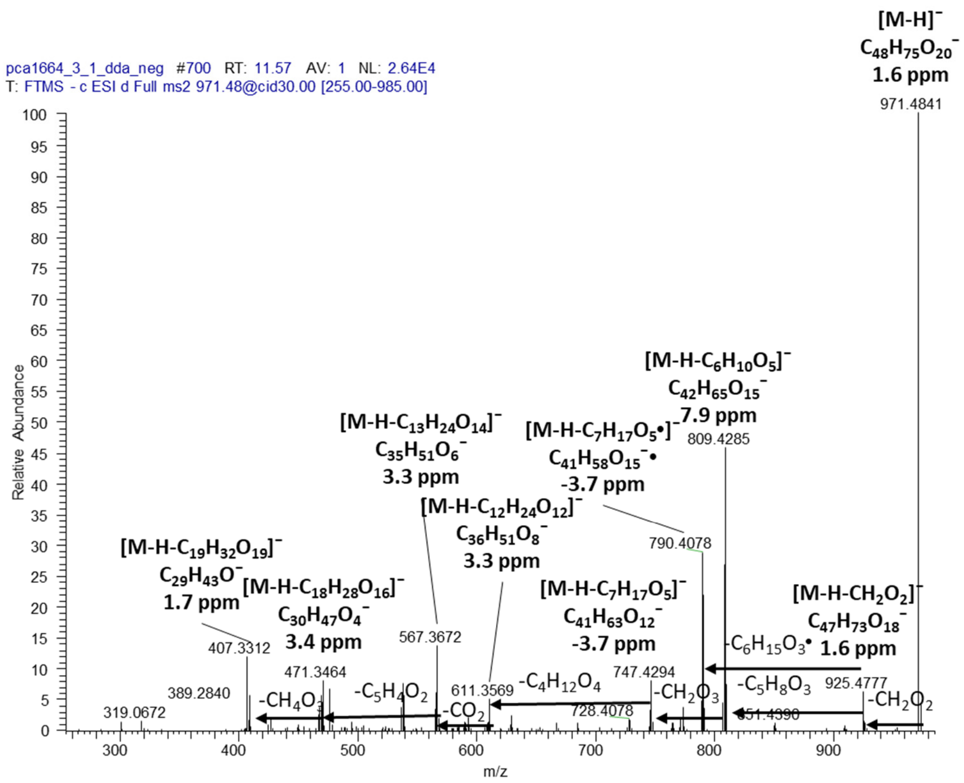

Figure S24. Tandem mass spectrum of *m/z* 971.48 at t_R 11.57 min corresponding to sophoraflavoside II isomer 3 (**21**), annotated in *Aralia elata var. mandshurica* (Rupr. & Maxim.) J. Wen extract. The spectrum was acquired with LIT-Orbitrap mass spectrometer operated in the negative product ion mode (collision energy 30 eV).

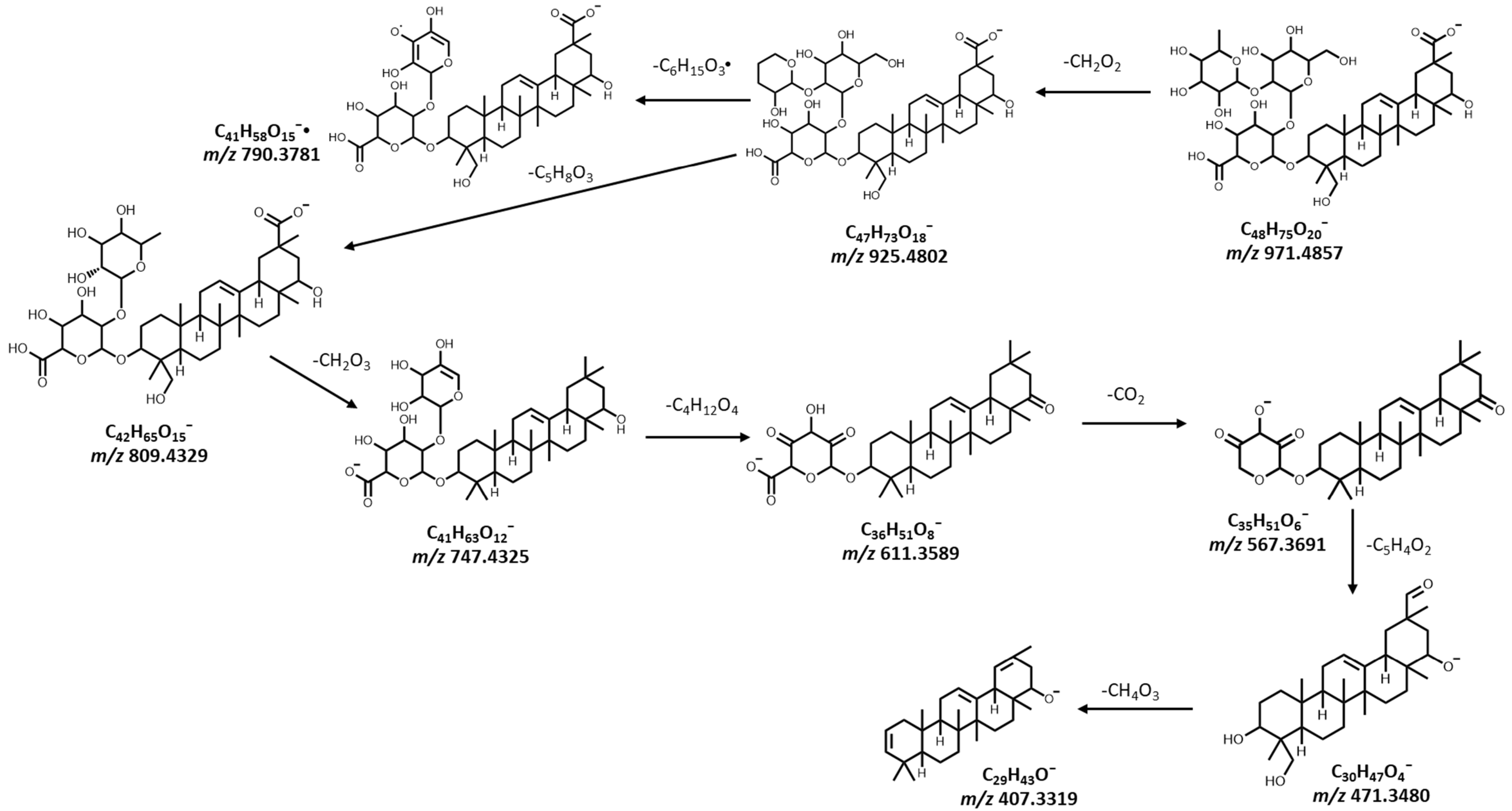

Figure S25. Proposed tandem mass spectrometric fragmentation patterns for the ion at *m/z* 971.48 ($[M-H]^-$, MS^2) corresponding to sophoraflavoside II isomer 3 (**21**).

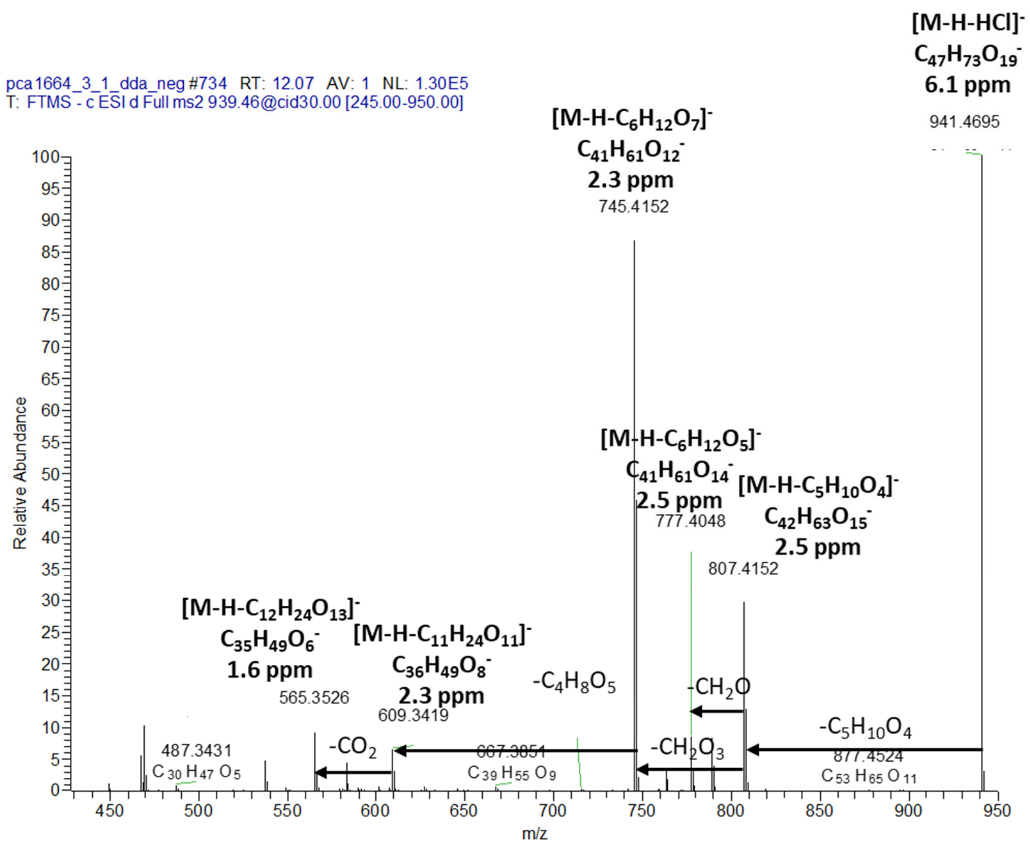

Figure S26. Tandem mass spectrum of *m/z* 939.46 at t_R 12.07 min corresponding to dihydrogypsogenin 3-O-pentopyranosyl-hexpyranoxyl-hexuronopyranoside isomer 2 (**22**), annotated in *Aralia elata var. mandshurica* (Rupr. & Maxim.) J. Wen extract. The spectrum was acquired with LIT-Orbitrap mass spectrometer operated in the negative product ion mode (collision energy 30 eV).

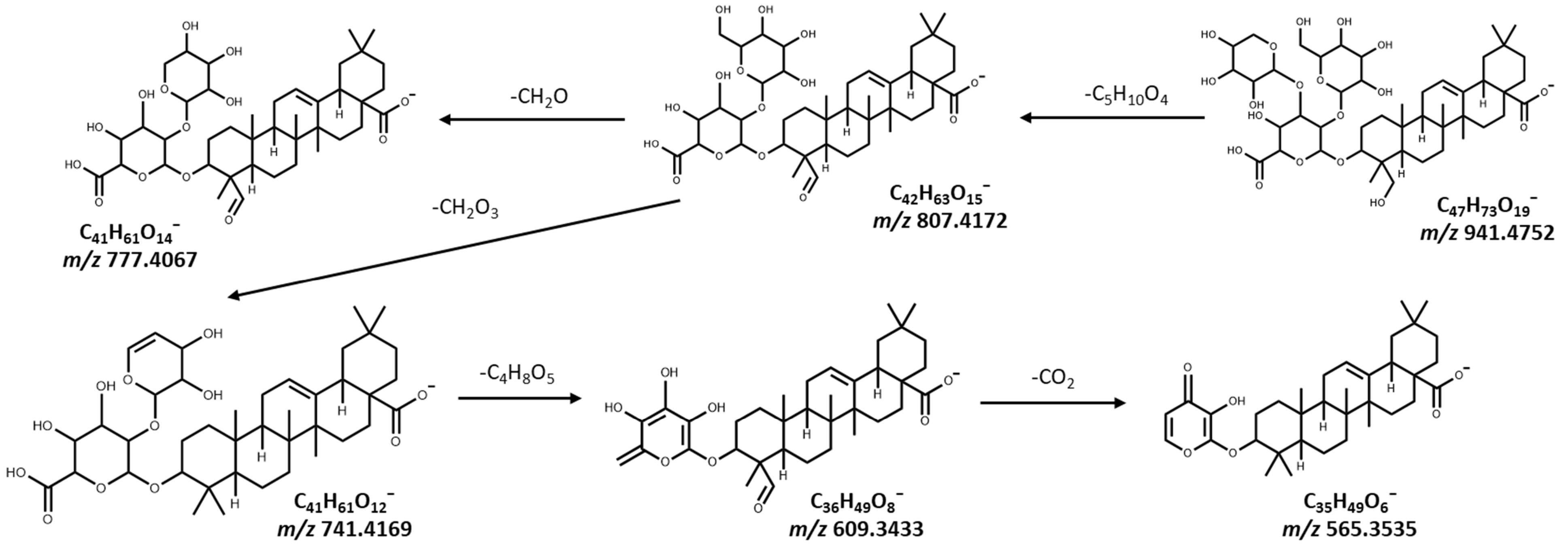

Figure S27. Proposed tandem mass spectrometric fragmentation patterns for the ion at *m/z* 939.46 ($[M-H]^-$, MS^2) corresponding to dihydrogypsogenin 3-O-pentopyranosyl-hexpyranoxyl-hexuronopyranoside isomer 2 (**22**).

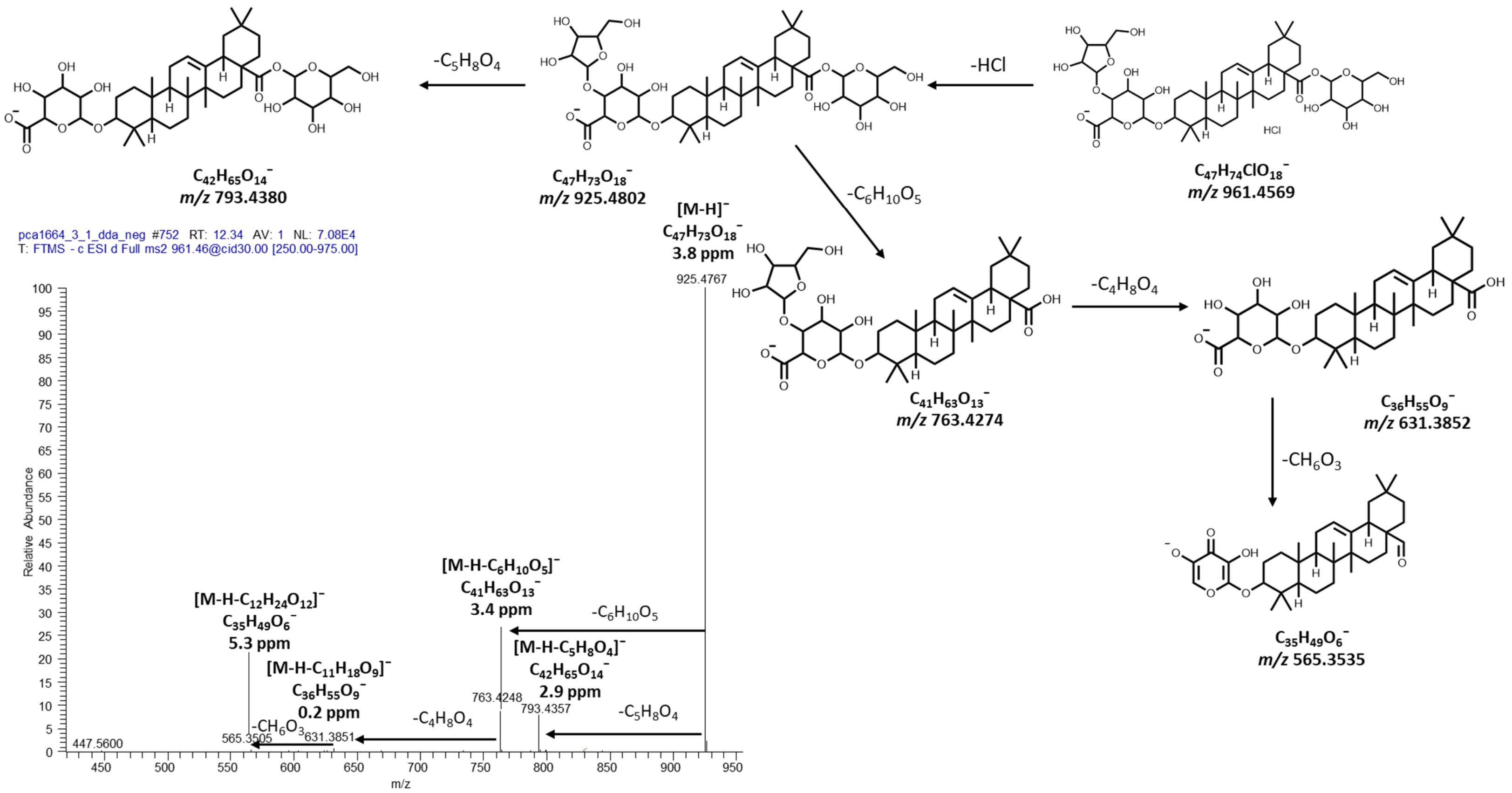

Figure S28. Tandem mass spectrum of *m/z* 961.46 at t_R 12.34 min corresponding to chikusetsusaponin IV (**23**), annotated in *Aralia elata var. mandshurica* (Rupr. & Maxim.) J. Wen extract. The spectrum was acquired with LIT-Orbitrap mass spectrometer operated in the negative product ion mode (collision energy 30 eV). Structures shown are suggestions.

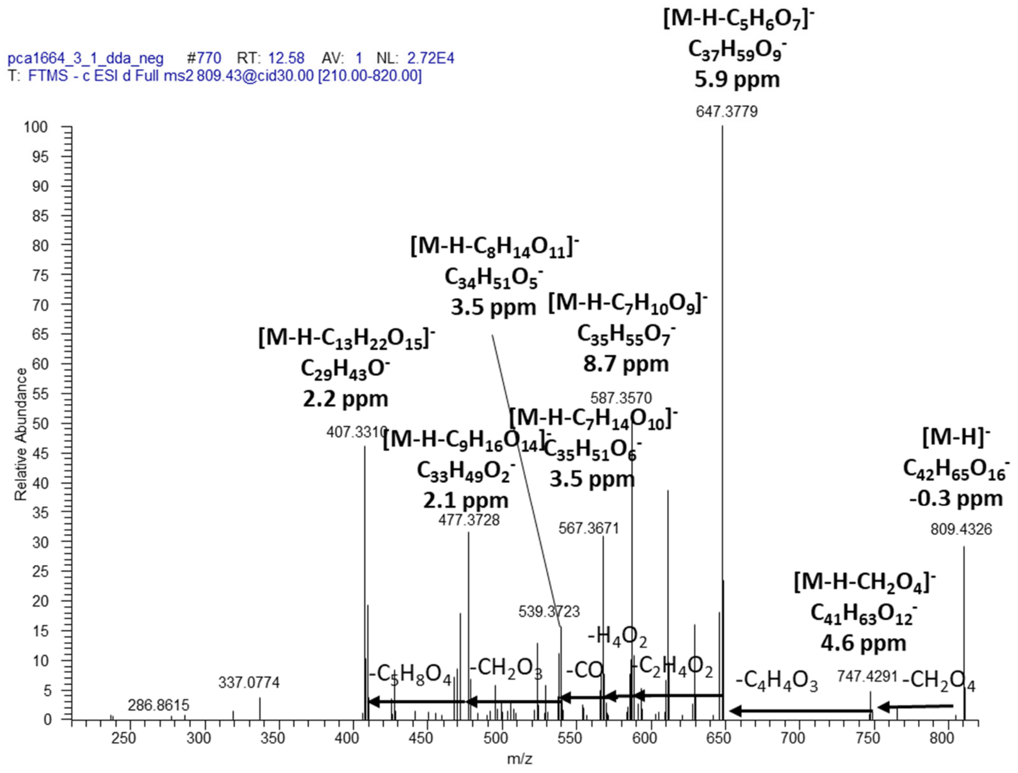

Figure S29. Tandem mass spectrum of *m/z* 809.43 at t_R 12.58 min corresponding to ilexoside XLVIII (**24**), annotated in *Aralia elata var. mandshurica* (Rupr. & Maxim.) J. Wen extract. The spectrum was acquired with LIT-Orbitrap mass spectrometer operated in the negative product ion mode (collision energy 30 eV).

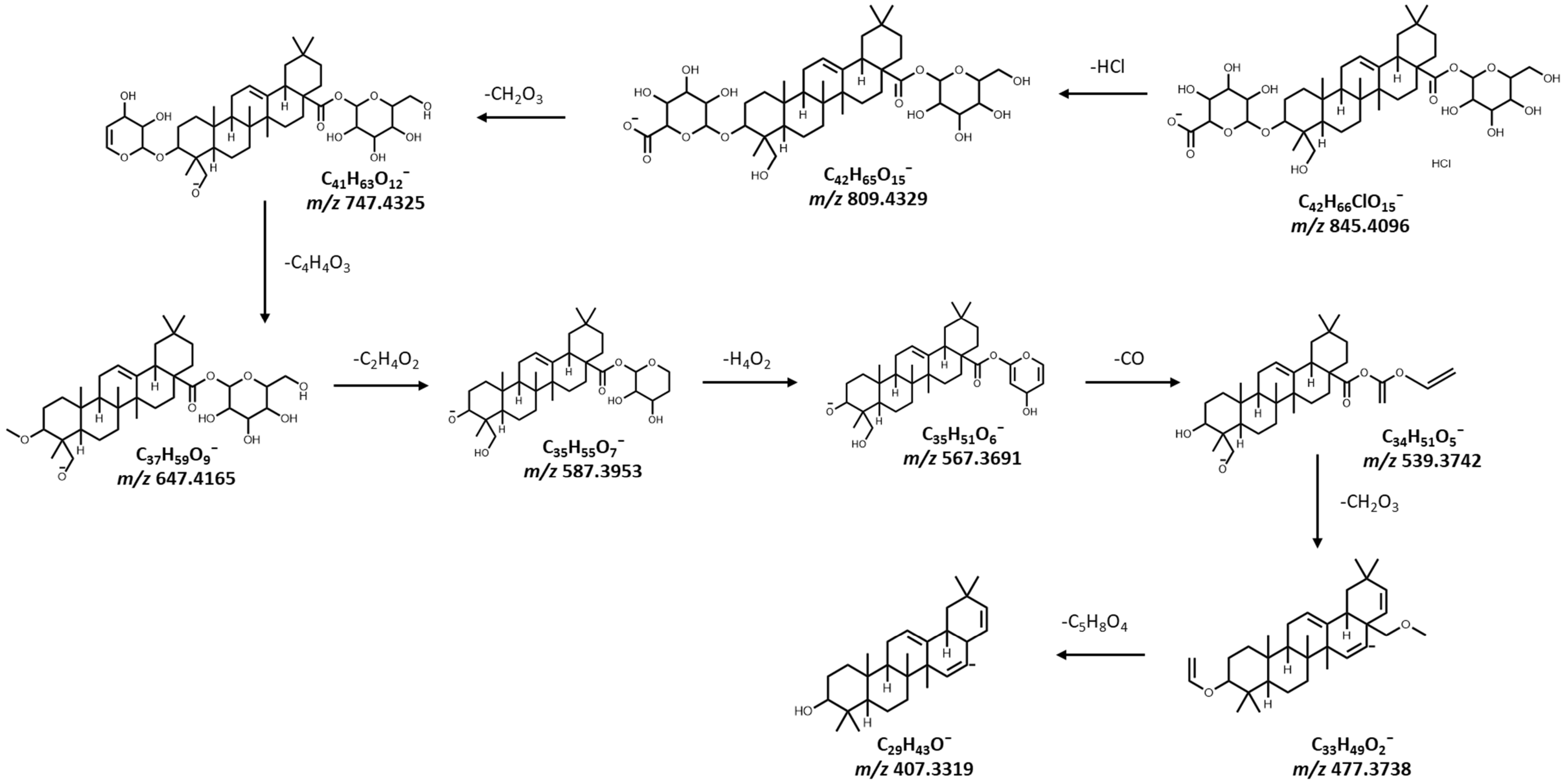

Figure S30. Proposed tandem mass spectrometric fragmentation patterns for the ion at *m/z* 809.43 ([MHCl-H]$^-$, MS^2) corresponding to ilexoside XLVIII (**24**).

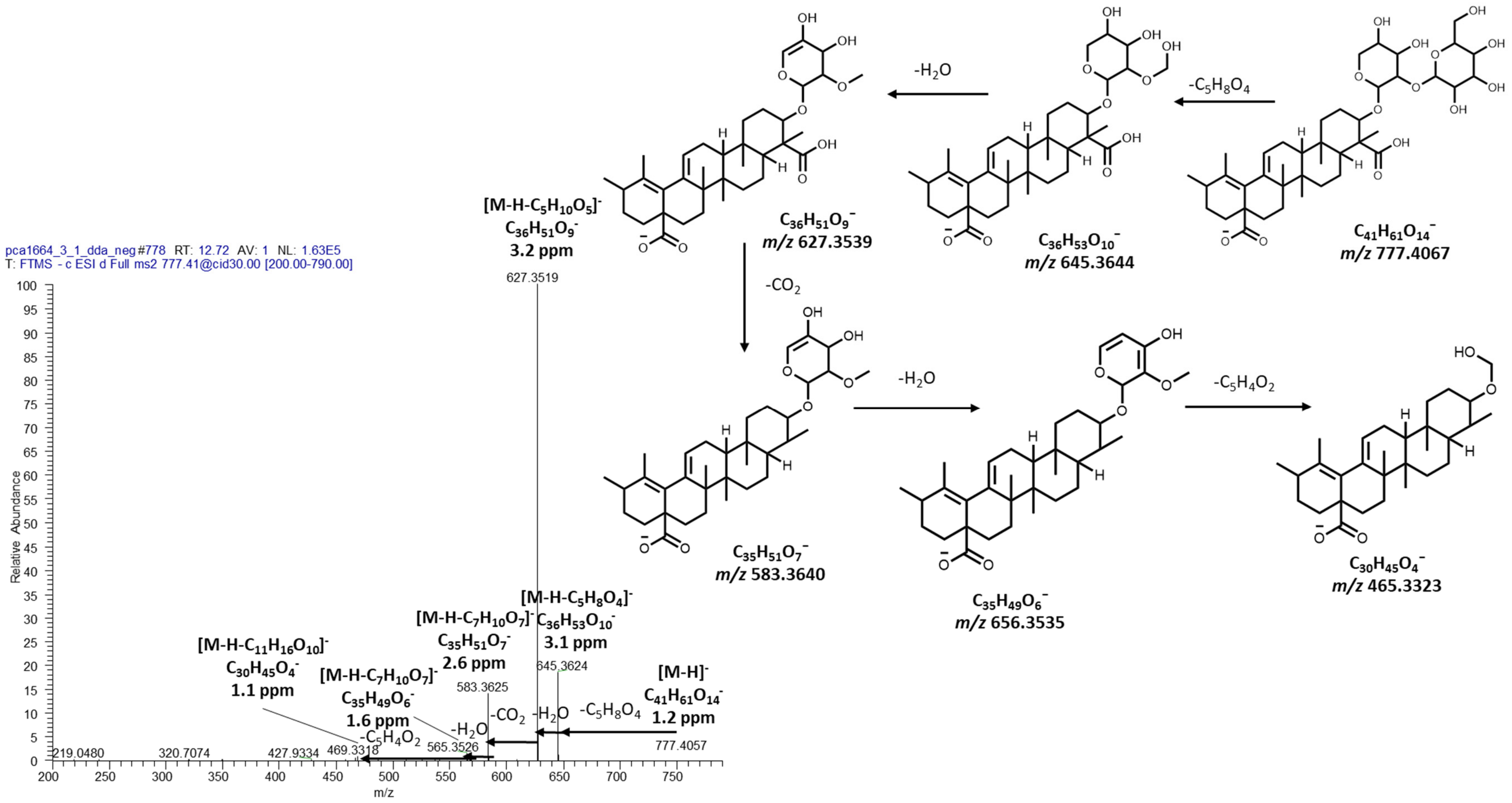

Figure S31. Tandem mass spectrum of *m/z* 777.41 at t_R 12.72 min corresponding to 3-*O*-hexopyranosyl-pentopyranosylurs-12,18-diene-24,28-dioic acid (**25**), annotated in *Aralia elata var. mandshurica* (Rupr. & Maxim.) J. Wen extract. The spectrum was acquired with LIT-Orbitrap mass spectrometer operated in the negative product ion mode (collision energy 30 eV). Structures shown are suggestions.

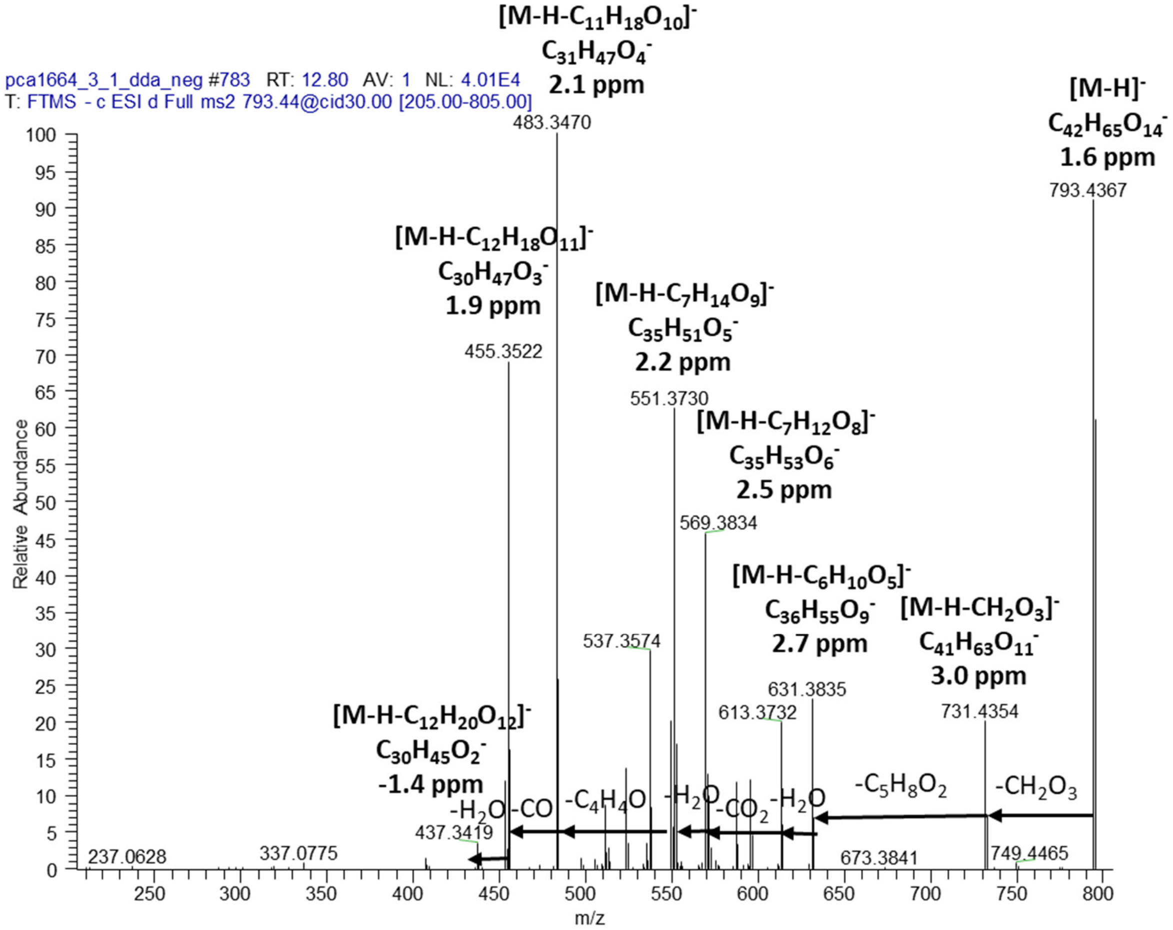

Figure S32. Tandem mass spectrum of *m/z* 793.44 at t_R 12.80 min corresponding to oleanolic acid-hexuronide-hexoside (**26**), annotated in *Aralia elata var. mandshurica* (Rupr. & Maxim.) J. Wen extract. The spectrum was acquired with LIT-Orbitrap mass spectrometer operated in the negative product ion mode (collision energy 30 eV).

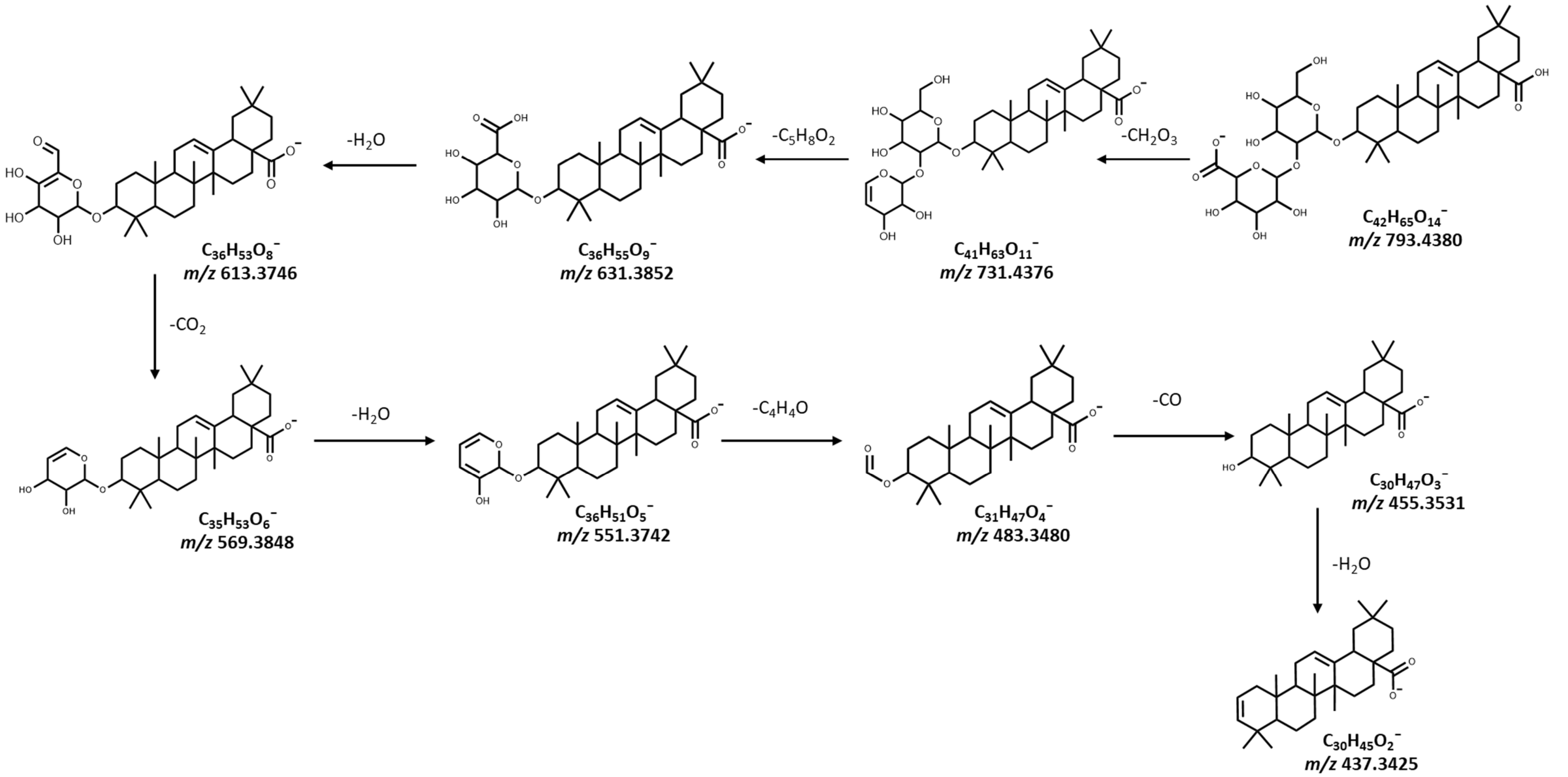

Figure S33. Proposed tandem mass spectrometric fragmentation patterns for the ion at *m/z* 793.44 ($[M-H]^-$, MS^2) corresponding to oleanolic acid-hexuronide-hexoside (**26**).

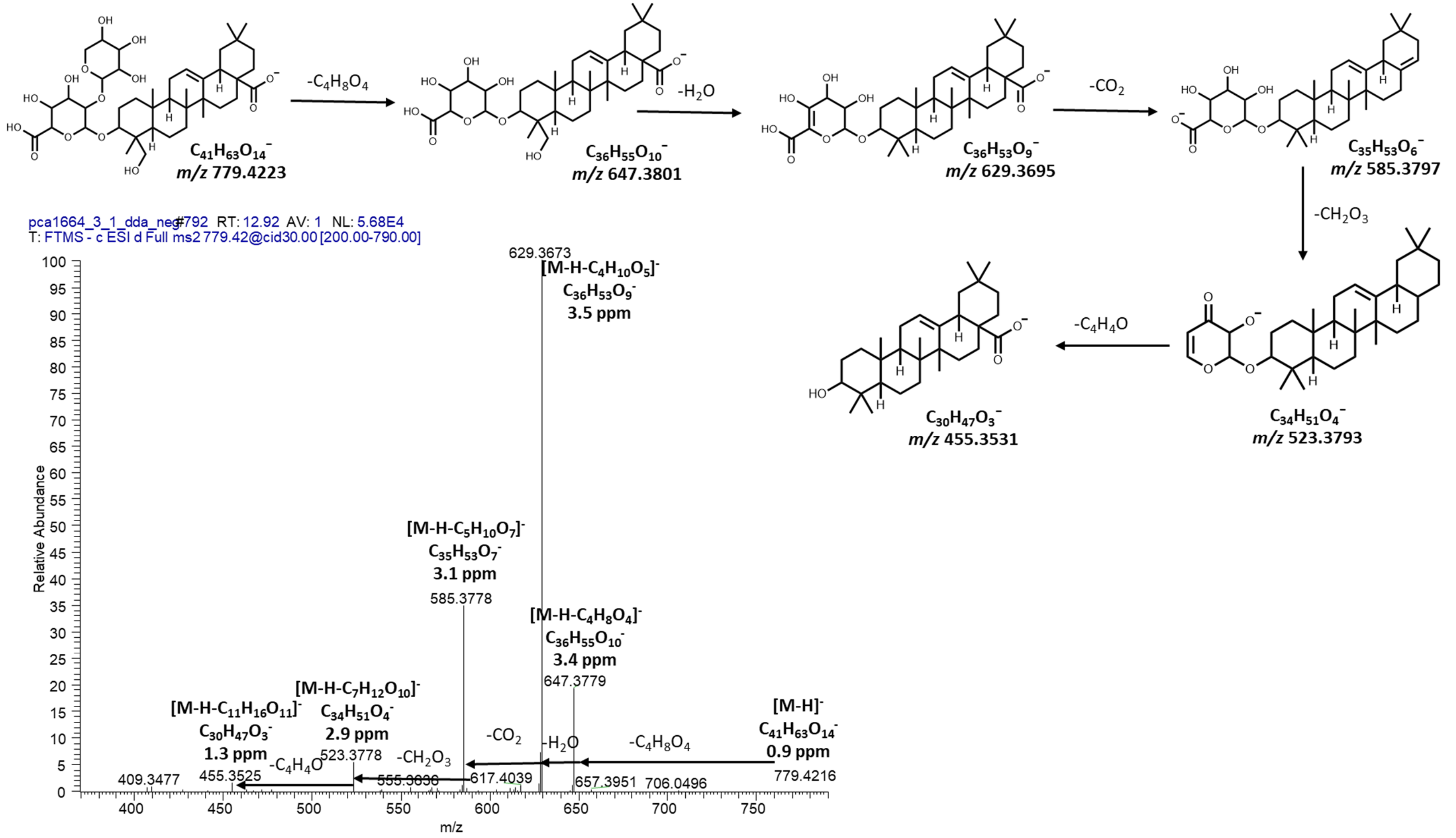

Figure S34. Tandem mass spectrum of *m/z* 779.42 at t_R 12.92 min corresponding to 3-*O*-[pentopyranosyl-hexuronopyranosyl] hederagenin (**27**), annotated in *Aralia elata var. mandshurica* (Rupr. & Maxim.) J. Wen extract. The spectrum was acquired with LIT-Orbitrap mass spectrometer operated in the negative product ion mode (collision energy 30 eV). Structures shown are suggestions.
